# Swine influenza A virus infection sets the local immunological landscape in subsequent infection with porcine reproductive and respiratory syndrome virus

**DOI:** 10.1186/s13567-025-01536-6

**Published:** 2025-06-08

**Authors:** Janaïna Grevelinger, Olivier Bourry, Selma Schmidt, François Meurens, Céline Deblanc, Caroline Hervet, Aline Perrin, Stéphane Gorin, Mireille Le Dimna, Stéphane Quéguiner, Thibaut Larcher, Patricia Renson, Frédéric Paboeuf, Wilhelm Gerner, Nicolas Bertho, Gaëlle Simon

**Affiliations:** 1 Oniris, INRAE, BIOEPAR, Nantes, France; 2 ANSES, Ploufragan-Plouzané-Niort Laboratory, Swine Virology Immunology Unit, Ploufragan, France; 3 The Pirbright Institute, Pirbright, Woking UK; 4 Research Group On Infectious Diseases in Production Animals (GREMIP) & Swine and Poultry Infectious Diseases Research Center (CRIPA), Faculty of Veterinary Medicine, University of Montreal, St-Hyacinthe, QC Canada; 5 Oniris, INRAE, APEX, Nantes, France; 6 ANSES, Ploufragan-Plouzané-Niort Laboratory, SPF Pig Production and Experimentation Unit, Ploufragan, France

**Keywords:** Influenza A virus, PRRSV, superinfection, local immune response, lung, dendritic cells, lymphocytes, NK, innate lymphoid cells

## Abstract

Farmed pigs are frequently exposed to respiratory infections, with swine influenza A virus (swIAV) and porcine reproductive and respiratory syndrome virus (PRRSV) being key drivers. Most co-infection studies with these viruses have focused on PRRSV infection followed by swIAV. However, the reverse scenario, where swIAV is given first and then PRRSV, has not been explored. This infection sequence is plausible under natural conditions and warrants further study, especially given that influenza A virus has been shown in mice to impair alveolar macrophages, which are the target cells for PRRSV. This study aimed to evaluate the impact of swIAV infection on the alveolar macrophage population, clinical signs, immune responses, and viral loads during a secondary infection with PRRSV initiated 7 days after the initial swIAV exposure. Results demonstrated that primary swIAV infection did not exacerbate the clinical progression of PRRSV infection, nor did it result in significant differences in PRRSV loads or affect the alveolar macrophage population in the lungs of super-infected pigs as compared to those of pigs infected with PRRSV alone. However, swIAV pre-infection was associated with an increase in the number of conventional dendritic cells type 1 (cDC1), perforin-expressing T cells and NK-related lymphocytes in bronchoalveolar lavage. This coincided with an increase of PRRSV-specific IFN-γ producing CD4 T cells in blood detected 7 days post-PRRSV infection. These findings suggest that a swIAV infection could enhance immune responses during subsequent PRRSV infection by recruiting cDC1 and inducing IL-12, promoting a type-1 immune response, highlighting the complex interplay and often unexpected outcomes of viral co-infections occurring in close temporal proximity.

**Supplementary Information:**

The online version contains supplementary material available at 10.1186/s13567-025-01536-6.

## Introduction

On farms, pigs are often exposed to multiple respiratory infections, which are frequently associated with complex respiratory diseases of viral and/or bacterial origin. This phenomenon is referred to as the porcine respiratory disease complex (PRDC) [1, 2]. Among the pathogens involved in PRDC, swine influenza A viruses (swIAV) and the porcine reproductive and respiratory syndrome virus (PRRSV) play a key role.

SwIAV belongs to the *Alphainfluenzavirus* genus in the *Orthomyxoviridae* family. These enveloped viruses have a segmented negative-sense single-stranded RNA genome. The main swIAV subtypes circulating in pig populations worldwide for decades include H1N1, H1N2, and H3N2 [3]. Swine influenza is a common respiratory disease in pig farms, causing clinical signs such as fever, breathing difficulties, coughing, and nasal discharge.

PRRSV, a positive-sense single-stranded RNA virus from the *Arteriviridae* family, is sub-divided into two species: PRRSV-1, mainly found in Europe and PRRSV-2, predominantly present in North America and Asia [4, 5]. This virus causes respiratory signs, reproductive disorders, and growth retardation and increased mortality in piglets. Its virulence varies depending on the strain and host characteristic [6].

Epidemiological studies conducted in France have shown a significant association between PRRSV and swIAV infections [7, 8]. Thus, more than half of the 125 farms studied in the Western part of the country displayed a positive correlation between antibodies against PRRSV-1 and the H1N2 subtype of swIAV [8]. This would indicate that in regions where these viruses are widespread, the risk of co-infection is particularly high.

Given the high risk of simultaneous infections in regions where both PRRSV and swIAV are prevalent, it is important to explore how these co-infections may affect animal health. Research has aimed to explore the interactions between swIAV and PRRSV through simultaneous or sequential infections, with PRRSV inoculated first, followed by swIAV a few days later. This was investigated in several studies, with outcomes varying depending on experimental conditions and virus strains. Some studies have reported aggravation of lung lesions [9, 10], while others found no differences compared to single virus infections [11–13]. Recent research also highlighted the complexity of immune responses triggered by co-infections. Bougon et al. [14] observed that a PRRSV-1 infection 8 days before exposure to swIAV H1N2 reduced influenza-like illness while increasing anti-swIAV antibodies and decreasing PRRSV-1 replication in lungs. Interferon-α (IFN-α) appeared to play a key role in the bidirectional interference between the two viral infections. Chrun et al. [13] showed that a co-infection with swIAV H3N2 and PRRSV-2 did not worsen clinical signs, reduced swIAV H3N2 viral load in the lungs, and even enhanced certain immune parameters, including PRRSV-2-specific CD8 T cell count and swIAV H3N2-specific IgG levels.

Most studies have focused on the inoculation of PRRSV followed by swIAV. Since influenza is an acute infection lasting no more than a week, whereas PRRS is an infection that can persist for months, it is more likely that animals infected with PRRSV will subsequently be co-infected with swIAV [2]. However, the alternative scenario, where animals are first infected with swIAV and secondly by PRRSV infection is plausible on pig farms, although it has never been explored and deserves further investigation.

In most cases, infection with influenza A virus (IAV) is reported to weaken the host, facilitating infections by opportunistic pathogens such as *Streptococcus suis* for instance in pigs [15] or *Streptococcus pneumoniae* in humans [16], ultimately worsening the overall illness. One proposed mechanism following investigations in mice was that influenza infection led to depletion of alveolar macrophages (AM), making the host more prone to secondary infections [17–19]. However, this theory is debated, as other studies in mice have shown AM persistence during influenza infection [18, 20]. Furthermore, respiratory viral infections may also have protective effects. Indeed, research in mice revealed that some respiratory viruses, including IAV, can induce a beneficial immune imprint in the lungs, offering better protection against subsequent bacterial infections, such as those caused by *Streptococcus pneumoniae* or *Escherichia coli* [21, 22].

In this context, the present study aimed to evaluate the impact of swIAV infection on the AM population and the early innate and adaptive immune responses during a PRRSV secondary infection. PRRSV virus was inoculated 1 week post-swIAV infection in order to avoid the initial interference caused by type I interferon (IFN-I) induction during the first 6 days post-swIAV infection, as IFN-I inhibits PRRSV replication [14]. By allowing IFN-I levels to return to baseline before PRRSV superinfection, we aimed to assess other potential effects of swIAV on PRRSV infection, particularly those related to immune cell function and composition.

We utilized two strains representative of viruses that were circulating on pig farms at the same period in Brittany, France.

## Materials and methods

### Virus strains

The swIAV strain A/swine/Ille et Vilaine/0415/2011 (H1_hu_N2, HA-clade 1B.1.2.3) and the so-called “Finistère” PRRSV strain (referenced as PRRS-FR-2005-29-24-1; PRRSV-1 subtype 1), both isolated from pig farms in Brittany, France, are part of the collections of the French Agency for Food, Environmental, and Occupational Health & Safety (ANSES, Ploufragan, France).

Swine IAV was propagated and titrated using Madin-Darby canine kidney (MDCK) cells (ATCC reference CCL-34), whereas primary porcine alveolar macrophages (AM) were used for PRRSV, as previously described [14]. AM were collected from SPF piglets free from CSFV, ASFV, PRV, FMDV, swIAV, TGEV, PPV, PRCV, PCV2, PRRSV, BDV, HEV, *Mycoplasma hyopneumoniae*, *Actinobacillus pleuropneumoniae*, *Pasteurella multocida*, *Bordetella bronchiseptica*, *Haemophilus parasuis*, *Streptococcus suis*. The PRRSV strain was propagated for three passages in AM and tested negative for bacteria (by bacteriology), mycoplasma, swIAV, CSFV, and PRV (by PCR).

### Animal experiment

**Figure 1:**
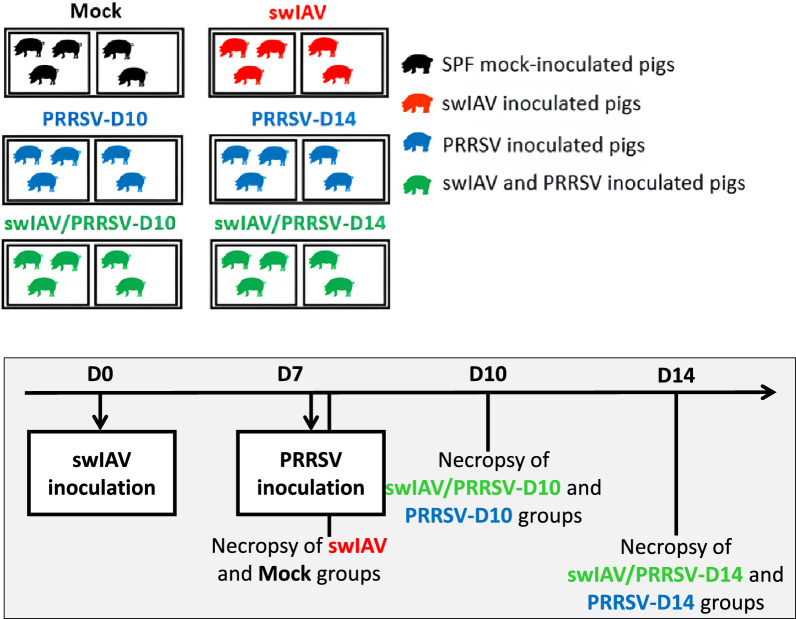
**Design of animal experiment**.

Thirty specific pathogen-free (SPF) Large White piglets from ANSES-Ploufragan’s protected animal facilities [23], were randomly assigned at 4 weeks of age to six groups based on parental origin, weight, and sex (Figure 1). The animals underwent a 7-week acclimatization period.

The experiment was conducted at ANSES facilities, which are accredited for animal research by the Direction Départementale de la Protection des Populations des Côtes d’Armor. The animal study received approval from the National Committee for Ethics in Animal Experimentation ANSES/ENVA/UPEC (approval no. 23-017 #40450) and authorization was granted by the French Ministry for Research (authorization no. APAFIS #40450-2023012311482365 v3).

The study was conducted on six groups of pigs, each receiving a specific inoculation scheme to assess the effect of swIAV infection on subsequent PRRSV infection. The experimental groups were as follows: Mock (control group, inoculated with culture medium (MEM) at D0 and euthanized at D7); swIAV (pigs inoculated with swIAV at D0 and euthanized at D7); swIAV/PRRSV-D10 and swIAV/PRRSV-D14 (pigs inoculated with swIAV at D0, then inoculated with PRRSV at D7, and euthanized either at D10 or at D14, equivalent to 3 or 7 days post-PRRSV inoculation, respectively); PRRSV-D10 and PRRSV-D14 (pigs inoculated with PRRSV at D7 and euthanized at D10 or D14, which means 3 or 7 days post-PRRSV inoculation, respectively).

The inoculations followed this protocol: On D0, pigs in the swIAV, swIAV/PRRSV-D10, and swIAV/PRRSV-D14 groups were intratracheally (IT) inoculated with 10^6^ TCID_50_ of swIAV in 5 mL per pig, as previously described by Bougon et al. [14]. The Mock, PRRSV-D10, and PRRSV-D14 groups received an equivalent volume of MEM at that time (D0). On D7, pigs in the swIAV/PRRSV-D10, swIAV/PRRSV-D14, PRRSV-D10, and PRRSV-D14 groups were intranasally (IN) inoculated with 5 × 10^5^ TCID_50_ of PRRSV in 5 mL per pig.

Rectal temperature, and clinical signs such as cough and sneezes were monitored daily. Hyperthermia was defined by a rectal temperature above 40 °C. Coughing and sneezing were recorded for 15 min in each room. Individual weights were measured weekly before the start of the experiment (before D0) and then twice a week from D0 onwards. Animals were also weighted just before PRRSV inoculation and prior to euthanasia. Food consumption at the room level was recorded daily.

Nasal swabs were collected in Virocult (MW915, Sigma Virocult®, MWE medical wire) at D-4, D2, D4, D7 (before euthanasia for the Mock and swIAV groups or before PRRSV inoculation), as well as on D10 and D14 before euthanasia. The supernatants were then frozen at −80 °C until RT-qPCR analysis. Animals were euthanized and necropsied according to the following schedule: on D7 for the swIAV and Mock groups, on D10 for the PRRSV-D10 and swIAV/PRRSV-D10 groups, and on D14 for the PRRSV-D14 and swIAV/PRRSV-D14 groups. After necropsies, bronchoalveolar lavages (BAL) were performed using sterile phosphate-buffered saline (PBS). A first BAL was performed with 40 mL of PBS, and the collected liquid was centrifuged at 500×*g* for 10 min at 4 °C to purify BAL fluid (BALF) from cells (BALC), and frozen at −20 °C for antibody and cytokine assays, and at −80 °C for RT-qPCR analyses. Two additional BALs with 100 mL of PBS each were carried out to collect and isolate more BALCs, which were stored in liquid nitrogen in fetal calf serum (FCS) with 10% dimethyl sulfoxide (DMSO) (Sigma-Aldrich). Lung tissue was taken from the right apical lobes, which were clamped during the BALs, and tracheobronchial lymph nodes (TBLN) were collected. These samples were frozen at −80 °C for RT-qPCR analyses or fixed in 4% formaldehyde (Merck) for histopathological analyses.

Serum samples were collected from clotted blood (centrifuged at 3000 × *g* for 5 min) on D-4, D2, D4, D7 (before euthanasia for the Mock and swIAV groups or before PRRSV inoculation), as well as on D8, and on D10 and D14 before euthanasia. Samples were then frozen at −20 °C for antibody and cytokine assays, and at −80 °C for RT-qPCR analyses. Peripheral blood mononuclear cells (PBMCs) were isolated from heparinized blood using Ficoll density gradient centrifugation with LeucoSep tubes (Greiner Bio One) and stored in liquid nitrogen in FCS with 10% DMSO.

### Histopathological examination of lungs

The formalin-fixed tissues were paraffin-embedded and sectioned into 4 µm slices. The sections were then stained with hematoxylin–eosin-saffron. Lesion intensity was evaluated based on parameters such as alveolar septal thickening, material accumulation in respiratory airways, inflammatory cell cuffing around bronchioles or vessels, proliferative bronchiolitis, alveolar emphysema, and bronchus-associated lymphoid tissue hyperplasia. Lesions were scored using two different scoring systems: one with three parameters from Jung et al. [24] and another composite system with three additional parameters from Larcher et al. [25]. For each system, scores were expressed as a percentage of the theoretical maximum.

### Viral quantifications

Total RNA was extracted from nasal swabs, serum, BALF, lung tissues, and lymph nodes using the ID Gene™ Mag Fast 384 Extraction Kit (Innovative Diagnostics) on the Auto-Pure 96 nucleic acid purification system (Allsheng).

For swIAV detection, duplex RT-qPCR targeting the M gene and β-actin was conducted using the Go Script RT mix for 1-Step RT-qPCR (Promega), following the method described by Cador et al. [26]. Quantification of the swIAV genome was achieved through serial dilutions of standardized M and β-actin mRNA, with results expressed as copy numbers of the M gene per 10^6^ copies of the β-actin gene.

PRRSV quantification was performed using duplex RT-qPCR with the SuperScript III Platinum one-step RT-qPCR kit (Life Technologies). Specific primers and probes targeting ORF5 of the “Finistère” strain and the porcine β-actin were used, as previously described [27]. Virus genome quantification was based on serial dilutions of standardized in vitro transcribed mRNA of the “Finistère” strain ORF5 RT-PCR amplicon (Eurofins genomics), with PRRSV genome levels reported as copy numbers per mL or per mg of sample.

### Cytokine quantifications

Cytokines in BALF, including GM-CSF, IFN-γ, IL-10, TNF-α, IL-1α, IL-1β, IL-6, IL-8, IL-18, and IL-12, were measured using a porcine-specific cytokine array (Milliplex PCYTMAG-23K, Merck Millipore) following the manufacturer’s instructions on the MAGPIX System (DiaSorin).

### Flow cytometry analysis

For mononuclear phagocyte cell staining in BALC a 5-color panel was applied (Table 1, “Myeloid cell panel”). Cells were thawed and plated in round-bottom 96-well microtiter plates with a staining buffer containing PBS, supplemented with 2 mM ethylenediaminetetraacetic acid (EDTA), 5% swine serum, and 5% goat serum. Surface staining was performed using primary mAbs against MHCII, CD172a, and CD11c. For secondary staining, rat anti-mouse-IgG2a-PE-Cy7 (Thermo Fisher Scientific), goat anti-mouse-IgG2b-APC-Cy7 (Abcam), and goat anti-mouse-IgG1-A647 (Thermo Fisher Scientific) were used to label MHCII, CD172a, and CD11c, respectively. Dead cells were identified using the LIVE/DEAD™ Fixable Aqua Dead Cell Stain Kit for 405 nm excitation (Thermo Fisher Scientific) after surface staining. Additional surface staining was performed with mAbs against CD1 and CD163. Staining steps were carried out at 4 °C for 30 min. Labelled cells were fixed with 1% paraformaldehyde (PFA) for 20 min at room temperature, followed by two washes with PBS. Samples were then acquired in PBS using a MACSQuant 10 cytometer (Miltenyi Biotec), equipped with 3 lasers (violet 405 nm, blue 488 nm, red 635 nm) and eight fluorescence detection channels. Data from 6 × 10^4^ to 4 × 10^5^ live cells per sample were recorded and analyzed using FlowJo software. Alveolar macrophages (AM), monocyte-derived dendritic cells (moDC), and conventional dendritic cells of types 1 and 2 (cDC1 and cDC2) were identified based on staining for MHC II, CD11c, CD163, CD172a, and CD1, as previously described. An anti-CD11c antibody was used to identify myeloid cells.

**Table 1:** **Primary antibodies used in flow cytometry analyses**

Antigen	Clone	Isotype	Conjugation	Work dilution	Supplier
Myeloid cell panel					
MHC-II	MSA3	Mouse IgG2a	Unconjugated	1/250	WSU
CD172a	74-22-15a	Mouse IgG2b	Unconjugated	1/500	WSU
CD11c	3A8	Mouse IgG1	Unconjugated	1/500	Homemade
CD1	76-7-4	Mouse IgG2a	FITC	1/25	Southern Biotech
CD163	2A10/11	Mouse IgG1	PE	1/25	Serotec
Treg panel					
CD3	BB23-8E6-8C8	Mouse IgG2a	PerCP-Cy5.5	1/10	BD Biosciences
CD4	74-12-4	Mouse IgG2b	Unconjugated	1/50	Southern Biotech
CD8α	76-2-11	Mouse IgG2a	Biotin	1/100	Southern Biotech
CD8β	PPT23	Mouse IgG1	PE	1/20	Bio-Rad
CD25	K231.3B2	Mouse IgG1	A647	1/10	Bio-Rad
Foxp3	FJK-16s	Rat IgG2a	eF450		Thermo Fisher Scientific
ICOS	C398.4A	Hamster IgG	BV605	1/20	BioLegend
Ki-67	B56	Mouse IgG1	BUV737	1/100	BD Biosciences
Unconventional T and NK cell panel					
TCR-γδ	PPT16	Mouse IgG2b	Unconjugated	1/400	The UK Immunological Toolbox
CD8α	76-2-11	Mouse IgG2a	Biotin	1/100	Southern Biotech
CD161	1D8/10E7	Mouse IgG1	Unconjugated	1/10	The UK Immunological Toolbox
CD3	BB23-8E6-8C8	Mouse IgG2a	PE-Cy7	1/200	BD Biosciences
CD8β	PPT23	Mouse IgG1	PE	1/20	Bio-Rad
CD16	G7	Mouse IgG1	FITC	1/17	Bio-Rad
NKp46	VIV-KM1	Mouse IgG1	A647	1/10	Bio-Rad
CD4	74-12-4	Mouse IgG2b	PerCP-Cy5.5	1/10	BD Biosciences
CD2	RPA-2.10	Mouse IgG1	BV605	1/20	BD Biosciences
T-bet	eBio4B10	Mouse IgG1	PE-Cy5	1/167	Thermo Fisher Scientific
Perforin	dG9	Mouse IgG2b	PE-Dazzle 594	1/4	BioLegend
PLZF	D-9	Mouse IgG1	A680	1/10	Santa Cruz Biotechnology
Intracellular cytokine panel					
CD4	74-12-4	Mouse IgG2b	purified	1/50	Southern Biotech
CD8α	76-2-11	Mouse IgG2a	Biotin	1/100	Southern Biotech
CD8β	PPT23	Mouse IgG1	PE	1/20	Bio-Rad
IFN-γ	P2G10	Mouse IgG1	A647	1/100	BD Bioscience
TNF-α	Mab11	Mouse IgG1	PE-Dazzle 594	1/10	BioLegend
IL-2	A150D3F1	Mouse IgG2a	PE/Cy7® (Abcam, ref: ab102903)^1^	1/160	Thermo Fisher Scientific

^1^Conjugation with conjugation kit (Abcam).

For the phenotyping of Tregs in BALC, an 8-color staining panel was applied (Table 1, “Treg panel”). Cells were thawed and seeded in round-bottom 96-well microtiter plates (Nunc MicroWell Plates, Thermo Fisher Scientific) with a staining buffer containing PBS and 10% heat-inactivated porcine plasma. Surface staining was performed using primary monoclonal antibodies (mAbs) against CD3, CD4, CD8α, CD8β, CD25, and ICOS (for details on antibodies, refer to Table 1). Secondary staining involved goat anti-mouse-IgG2b-A488 (Jackson Immuno Research) and Streptavidin-BV650 (BioLegend) for labeling of CD4 and CD8α, respectively. Dead cells were marked with Fixable Viability Dye eFluor780 (Thermo Fisher Scientific) following the manufacturer’s guidelines. Cells were then fixed and permeabilized using the eBioscience™ Foxp3/Transcription Factor Staining Buffer Set (Thermo Fisher Scientific). Intracellular staining was performed with mAbs against Foxp3 and Ki-67. Surface staining was carried out at 4 °C for 20 min and intracellular staining at 4 °C for 30 min. After intracellular staining, cells were washed twice and resuspended in Perm/Wash Buffer for analysis.

A second, 12-color antibody panel was applied for characterisation of unconventional T cells and NK cells (Table 1, “unconventional T and NK cell panel”). Here, antibodies against the surface molecules TCR-γδ, CD8α, and CD161 were used. Secondary staining included rat anti-mouse-IgG2b-BUV395 (BD Biosciences), streptavidin-BV650 (BioLegend), and goat anti-mouse-IgG1-BV421 (Jackson Immuno Research) to label TCR-γδ, CD8α, and CD161, respectively. Additional surface staining was performed with mAbs against CD3, CD8β, CD16, NKp46, CD4, and CD2. The eBioscience™ Foxp3/Transcription Factor Staining Buffer Set (Thermo Fisher Scientific) was used for fixation and permeabilization as described previously. Intracellular staining was performed with mAbs against T-bet, perforin, and PLZF. Samples from both panels were analyzed on a Cytek Aurora spectral cytometer (Cytek Biosciences) with 5 lasers (UV 355 nm, violet 405 nm, blue 488 nm, yellow-green 561 nm, red 640 nm) and 64 fluorescence detection channels. Spectral unmixing was performed using SpectroFlo software (version 3.2.1, Cytek Biosciences) with single-stained reference samples. Autofluorescence signatures were subtracted using unstained controls. Data from 2 × 10^4^ to 3 × 10^5^ live lymphocytes per sample were recorded and analyzed using FlowJo software for Windows (version 10.9.0, BD Biosciences).

### Intracellular cytokine staining (ICS)

For ICS a 6-color staining panel was applied (Table 1, “Intracellular cytokine panel”). PBMCs were thawed and plated in round-bottom 96-well microtiter plates with 5 × 10^5^ cells per well, in a final volume of 200 μL per well. Cells were cultivated in Roswell Park Memorial Institute (RPMI) 1640 (Sigma-Aldrich) supplemented with 1% penicillin (100 IU/mL) and streptomycin (100 μg/mL) (PS) (Gibco, Thermo Fisher Scientific) and 10% fetal calf serum (FCS) (Life Science Production). They were exposed to either PRRSV-1 (“Finistère” strain) at a multiplicity of infection (MOI) of 1 for 18 h, phorbol 12-myristate 13-acetate (PMA) (50 ng/mL) and ionomycin (500 ng/mL) as a positive control for the final 4 h, or cell culture medium alone. Brefeldin A (BD GolgiPlug™, BD Biosciences) was added to all conditions for the final 4 h at a concentration of 1 μg/mL. After incubation, cells were harvested, centrifuged, and resuspended in staining buffer containing PBS with 3% FCS. Cells were then surface-stained with mAbs against CD4, CD8α, and CD8β. Subsequently, goat-anti-mouse-IgG2b-A488 (Jackson Immuno Research) and Streptavidin-BV421 (BioLegend) were used to label CD4 and CD8α, respectively. Dead cells were identified using Fixable Viability Dye eFluor780 (Thermo Fisher Scientific) after surface staining. Cells were then fixed and permeabilized using the BD Cytofix/Cytoperm™ Fixation/Permeabilization Kit (BD Biosciences). Intracellular staining was performed with mAbs against IFN-γ, IL-2 and TNF-α. Incubation steps were performed in the same way as described above. After intracellular staining, cells were washed twice and resuspended in Perm/Wash Buffer (BD Biosciences). Samples were acquired on the Cytek Aurora cytometer. Data from a minimum of 4 × 10^5^ live lymphocytes per sample were recorded and analyzed using FlowJo software. Data for TNF-α labelling was not analyzed due to a technical issue.

### Antibody assessment in sera and BALF

Anti-swIAV (NP protein) IgG was detected using the ID Screen Influenza A Nucleoprotein Swine Indirect kit (Innovative Diagnostics) in serum at a 1:100 dilution and in BALF at a 1:2 dilution. Anti-swIAV IgA was measured in BALF at a 1:2 dilution using the same kit, with a modified protocol using a goat anti-pig IgA antibody HRP conjugate (Euromedex) at a 1:3000 dilution and in-house controls to calculate sample-to-positive (S/P) ratios.

Anti-PRRSV (N protein) immunoglobulin G (IgG) was measured in serum and BALF using the IDEXX PRRS X3 ELISA kit (IDEXX Laboratories). In serum, the kit was used according to the manufacturer’s instructions at a 1:40 dilution, while in BALF, an adapted protocol was applied with a 1:2 dilution of the samples. For detecting anti-PRRSV immunoglobulin A (IgA) in 1:2 diluted BALF and anti-PRRSV immunoglobulin M (IgM) in 1:40 diluted serum, the same kit was employed replacing the anti-pig IgG conjugated antibody by a goat anti-pig IgA or a goat anti-pig IgM HRP conjugated antibody (Euromedex) at a 1:3000 or 1:25 000 dilution, respectively. For anti-PRRSV IgA or IgM assays, in-house calibrated negative and positive BALF or serum controls were used to calculate sample-to-positive (S/P) ratios.

### Statistical analyses

**Figure 2:**
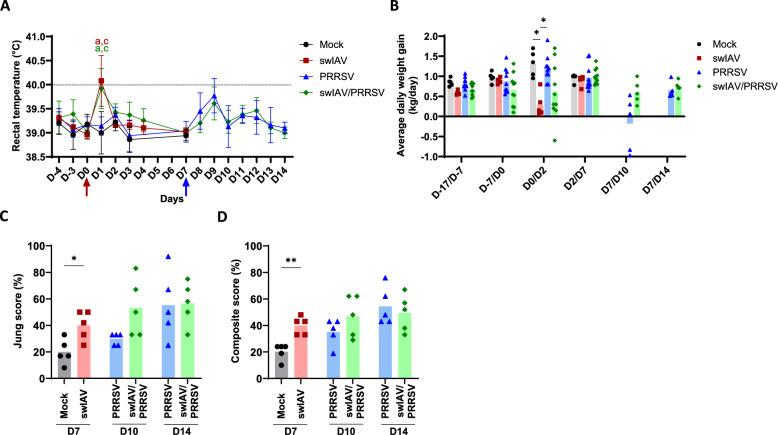
**Clinical signs and lung lesions. A** Rectal temperature. On D0, the red arrow indicates swIAV inoculation, and on D7, the blue arrow indicates PRRSV inoculation. Statistical analysis was performed using the Kruskal–Wallis unpaired, non-parametric test. Different letters (a-d) indicate that the considered group (specified by its color) was significantly different from the Mock group (a), from the swIAV group (b), from the PRRSV group (c) or from the swIAV/PRRSV group (d) with *p* < 0.05 (mean ± SD; *n* = 5–10). **B** Average daily weight gain (mean; *n* = 5–10). Statistical analysis was performed using the Kruskal–Wallis unpaired, non-parametric test, (*) *p* < 0.05. **C**, **D** Lung sections were evaluated for histopathological lesions. **C** Jung score and **D** Composite score (mean; *n* = 5). Statistical analysis was performed using the Mann–Whitney unpaired, non-parametric test, (*) *p* < 0.05 or (**) *p* < 0.01.

**Figure 3:**
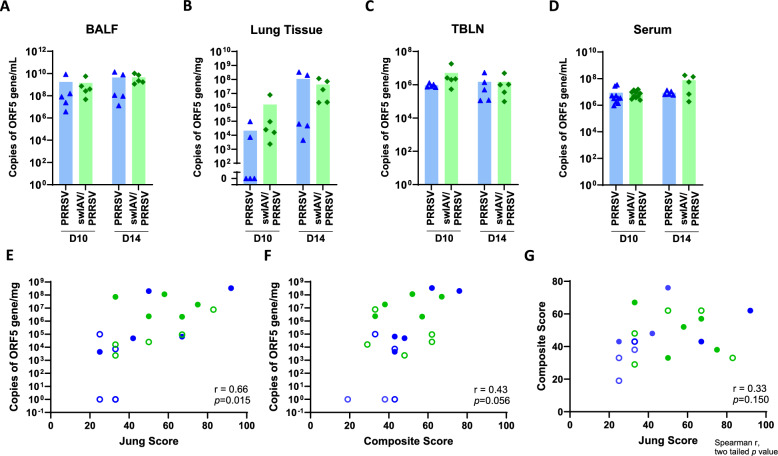
**PRRSV load in BALF, lung tissue, tracheobronchial lymph node and serum.** **A**–**D** Quantification of PRRSV loads by RT-qPCR in **A** BALF, **B** lung tissue, **C** tracheobronchial lymph nodes, and **D** serum. Statistical analysis was performed using the Mann–Whitney unpaired, non-parametric test (mean; *n* = 5–10). **E**, **F**, **G** Lung lesion scores and PRRSV lung load correlations. Statistical analysis was performed using the Spearman two tailed correlation test.

**Figure 4:**
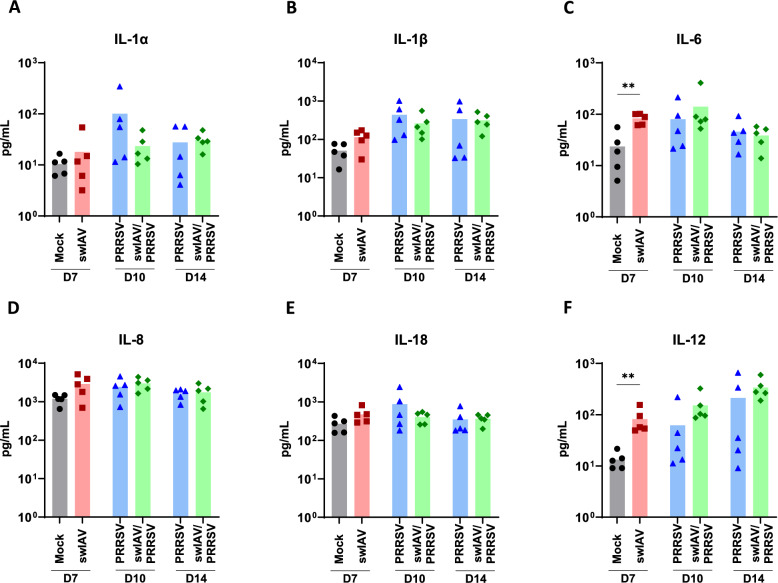
**Cytokine profile in BALF.** **A**–**F** Cytokine levels were quantified in BALF using multiplex immunoassays. **A** IL-1α, **B** IL-1β, **C** IL-8, **D** IL-18, **E** IL-6 and **F** IL-12. Statistical analysis was performed using the Mann–Whitney unpaired, non-parametric test, (*) *p* < 0.05 or (**) *p* < 0.01 (mean; *n* = 5).

**Figure 5:**
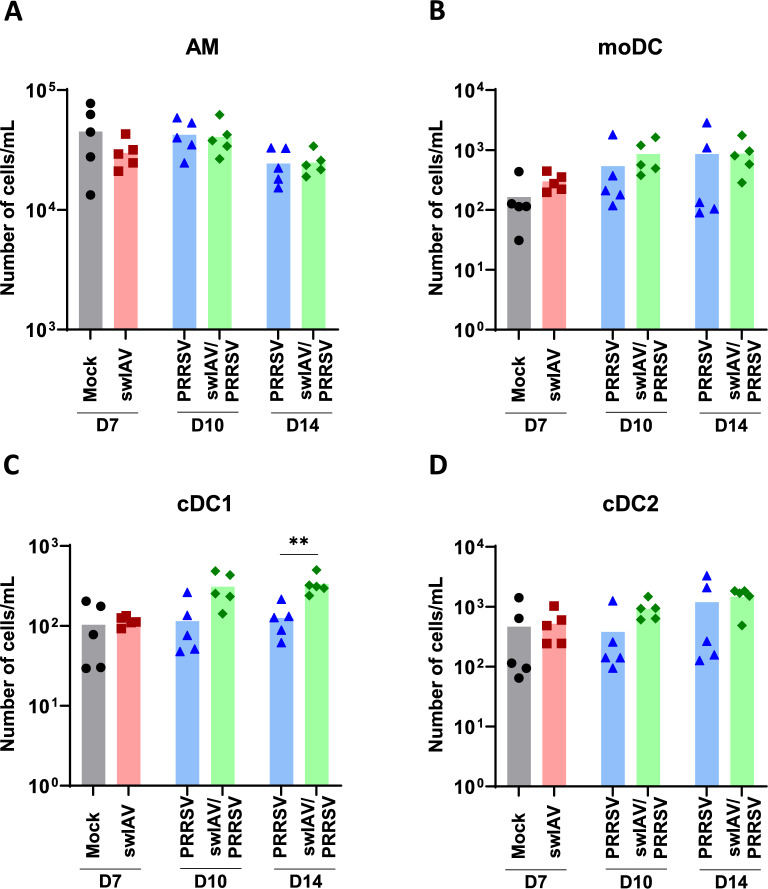
**Absolute numbers of myeloid cell populations in the BALC.** **A**–**D** The absolute numbers of myeloid cells were determined in the BALC using flow cytometry. **A** Alveolar macrophages (AM), **B** monocyte-derived dendritic cells (moDC), **C** conventional type 1 dendritic cells (cDC1), and **D** conventional type 2 dendritic cells (cDC2). Statistical analysis was performed using the Mann–Whitney unpaired, non-parametric test, (*) *p* < 0.05 or (**) *p* < 0.01 (mean; *n* = 5).

**Figure 6:**
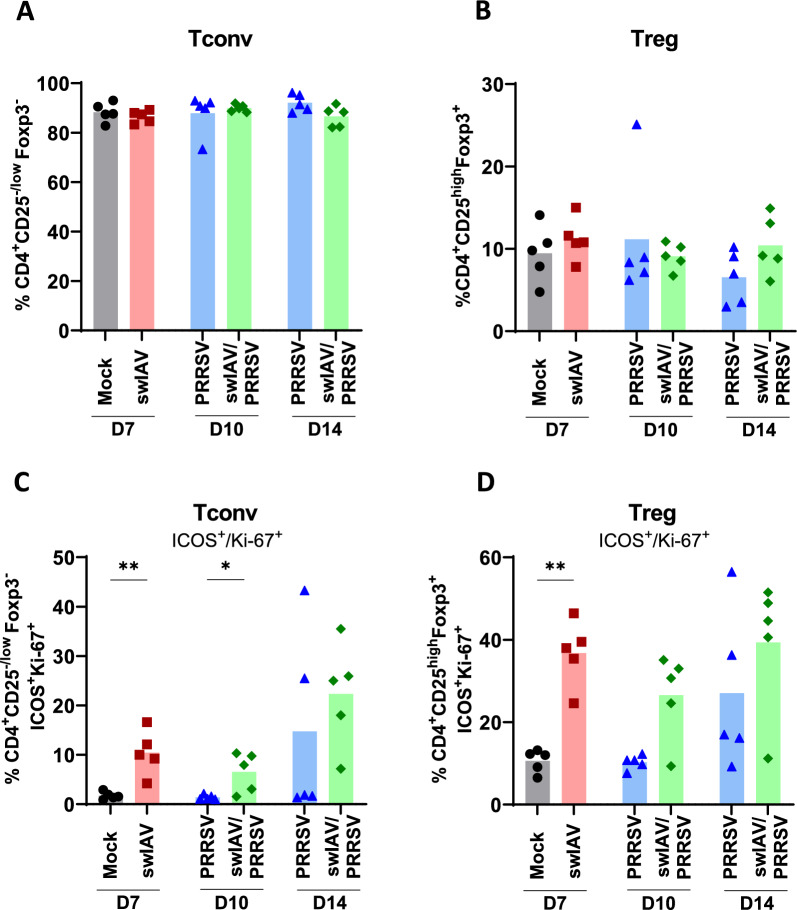
**Proportions of regulatory and conventional CD4 T cells in BALC.** **A**–**D** The percentages of Tconv and Treg CD4 cells in the BALC were determined using flow cytometry. (A, B) presents the percentages within total CD3^+^CD4^+^ cells. **A** Shows CD4^+^CD25^−/low^Foxp3^−^ Tconv cells. **B** Shows CD4^+^CD25^high^Foxp3^+^ Treg cells. **C**, **D** Shows the percentages of ICOS^+^Ki-67^+^ within the respective parental population of **C** Tconv and **D** Treg. Statistical analysis was performed using the Mann–Whitney unpaired, non-parametric test, (*) *p* < 0.05 or (**) *p* < 0.01 (mean; *n* = 5).

**Figure 7:**
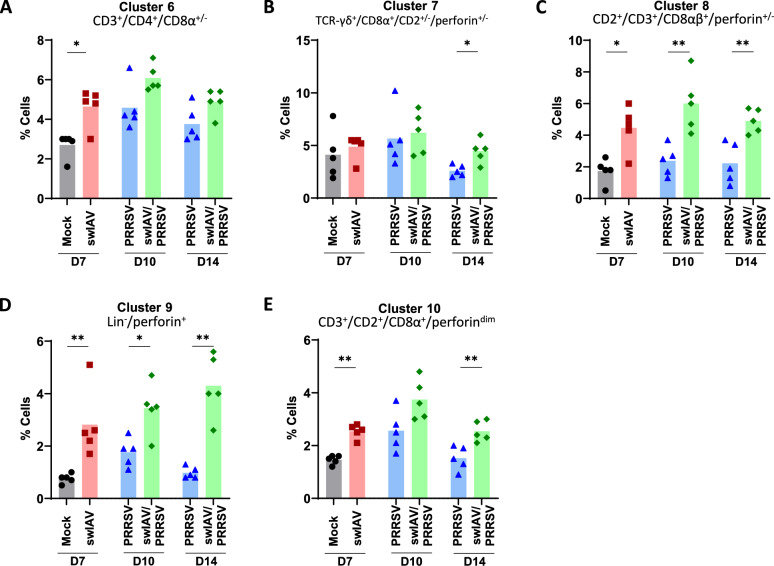
**Proportions of lymphoid cell populations in the BALC.** **A**–**E** Lymphocyte populations were clustered using the t-SNE algorithm to analyse flow cytometry data, with clusters 6 to 10 being presented. The t-SNE algorithm is based on live lymphocytes. Statistical analysis was performed using the Mann–Whitney unpaired, non-parametric test, (*) *p* < 0.05 or (**) *p* < 0.01 (mean; *n* = 5).

**Figure 8:**
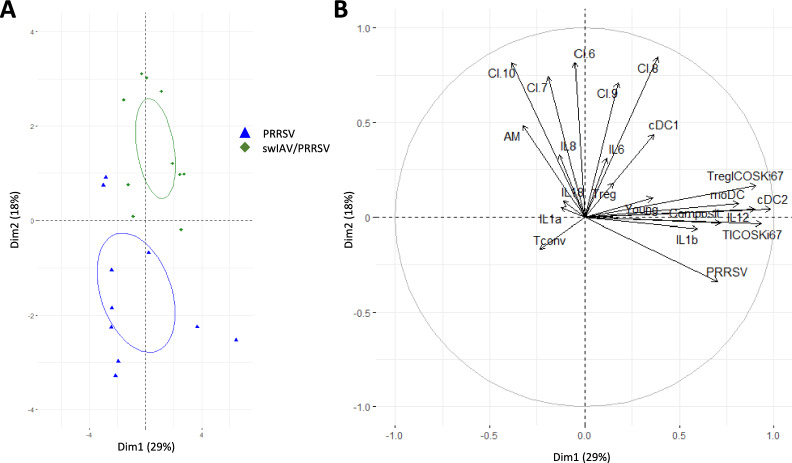
**Principal component analysis of swIAV/PRRSV and PRRSV infected groups.** PCA includes PRRSV loads in the lung (Figure 3B) along with all the other variables depicted in Figures 4, 5, 6, 7, encompassing the data from PRRSV-infected and swIAV/PRRSV-infected animals (D10 PRRSV, D10 swIAV/PRRSV, D14 PRRSV, D14 swIAV/PRRSV groups). The first two PCA dimensions (Dim) accounted for 29% (Dim1) and 18% (Dim2) of the data complexity respectively. **A** Graph of individuals, each symbol depicts one animal from PRRSV groups (blue triangles) or swIAV/PRRSV groups (green lozenges). Confidence ellipses define the regions that contains 95% of the samples from each condition (PRRSV or swIAV/PRRSV) that can be drawn from the underlying Gaussian distribution. **B** Projections of the 22 variables (Composit score, Young score; PRRSV Lung load; serum IL-1α, IL-1β, IL-6, IL-8, IL-12, IL-18 concentrations; BALC content in AM, moDC, cDC1, cDC2, Tconv, Treg, TICOSKi67, TregICOSKi67, Cl.6, Cl.7, Cl.8, Cl.9, Cl.10) on the two first dimensions.

Non-normal distribution of the data was determined by the Shapiro–Wilk test. The Kruskal–Wallis test followed by Dunn’s correction was used for multiple unpaired comparisons among four groups, and significance levels were directly indicated in Figure 2A and B (rectal temperature and average daily weight gain data). Unpaired comparisons between two groups (Mann–Whitney test) were applied for Figures 2C, 3, 4, 5, 6, 7, 8 (histopathological, virological, and immunological data). The Mann–Whitney test was used to compare Mock vs. swIAV, PRRSV-D10 vs*.* swIAV/PRRSV-D10, and PRRSV-D14 *vs*. swIAV/PRRSV-D14. However, Kruskal–Wallis test was also applied for unpaired comparisons among four groups on these data, and significances are depicted in Additional file 1.

The absolute number of mononuclear phagocyte cells was calculated by determining the percentage of live cells using flow cytometry, then multiplying this percentage by the total number of BALC counted, all divided by 100 and multiplied by the volume of BALF collected.

The script used for high-dimensional flow cytometry analysis was developed by Adrian Liston’s group (The Babraham Institute, UK) and is available on GitHub [28]. t-SNE plots were generated using R (version 4.3.0). t-SNE algorithm was run on live lymphocytes using the parameters CD3, CD4, CD8α, CD8β, CD16, NKp46, TCR-γδ, CD2, CD161, Perforin, PLZF and T-bet. Samples of five pigs per treatment group (Mock, swIAV, PRRSV-D10, PRRSV-D14, swIAV/PRRSV-D10, swIAV/PRRSV-D14) were used with 5000 cells per sample and 5000 iterations per run. Statistical analyses were performed using GraphPad Prism Software (version 10.2.3).

Principal component analysis (PCA) was performed using the FactoMineR package of RStudio (version 3.6.1) in order to investigate the relation between PRRSV loads in the lung (Figure 3B) along with all the other variables depicted in Figures 4, 5, 6, 7, encompassing the data from PRRSV-infected and swIAV/PRRSV-infected animals (D10 PRRSV, D10 swIAV/PRRSV, D14 PRRSV, D14 swIAV/PRRSV groups).

## Results

### Pre-infection with swIAV did not affect the clinical progression of PRRS

To compare the clinical outcomes of a single PRRSV inoculation to successive inoculations of swIAV and PRRSV 7 days apart (Figure 1), rectal temperature and respiratory signs (coughing, sneezing, and breathing rate) were monitored daily. On D1, the animals that received swIAV intratracheally showed hyperthermia (rectal temperature > 40 °C) in four out of five animals in the swIAV group and in five out of ten animals in the swIAV/PRRSV group (mean 40.1 ± 0.5 °C and 39.9 ± 0.4 °C, respectively). These temperatures were significantly higher than those in the Mock and PRRSV groups (*p* < 0.05) (Figure 2A). By D2, the animals’ temperatures returned to normal (below 40 °C). On D9, 2 days after PRRSV inoculation, four out of ten animals in the PRRSV group (mean 39.8 ± 0.4 °C) and two out of ten in the swIAV/PRRSV group (mean 39.6 ± 0.3 °C) showed an increase in temperature above 40 °C. There was no significant difference between the PRRSV and swIAV/PRRSV groups.

Some animals showed respiratory signs. On the first day after swIAV inoculation, one out of fifteen animals was coughing. By D9 (2 days after PRRSV inoculation), one out of ten animals in the swIAV/PRRSV group was sneezing, and the same animal was coughing on D10 (3 days after PRRSV infection). By D10, two out of ten sequentially inoculated animals showed respiratory signs (one was coughing and the other sneezing), compared to one out of ten in the PRRSV-only group, which exhibited a cough.

Average daily weight gain (ADWG) was significantly decreased in the swIAV group between D0 and D2, compared to the Mock and PRRSV groups (*p* < 0.05). However, in the swIAV/PRRSV group, no significant difference was observed between this group and the Mock and PRRSV groups. After PRRSV inoculation, there was no significant growth difference between the PRRSV group and the swIAV/PRRSV group (Figure 2B).

Microscopic examination of the lung tissue revealed that, compared to control animals, the alveolar walls were significantly thickened in all infected animals, regardless of single or super-infections, that corresponds to interstitial pneumonia. Additionally, the respiratory airways were occluded by necrotic debris and inflammatory cells. Perivascular cuffing by inflammatory cells was observed in the most severely affected animals. The severity of lung lesions was assessed using two different scoring systems (Jung score and Composite score), both of which gave similar results (Figures 2C, D). By D7 post-swIAV inoculation, significant lung lesions were observed in the swIAV group compared to the Mock group (*p* < 0.05). After PRRSV inoculation, no differences were observed on D10 and D14 between the PRRSV group and the swIAV/PRRSV group.

Overall, these results suggested that the initial swIAV infection did not worsen the clinical signs and lung lesions caused by PRRSV.

### Pre-infection with swIAV did not affect PRRSV loads

Using RT-qPCR, the viral loads of PRRSV and swIAV genomes were monitored in BALF, lung tissue, tracheobronchial lymph nodes, nasal swab supernatants (swIAV) and serum samples (PRRSV) from all groups. Neither PRRSV nor swIAV genome was detected in the Mock-inoculated group.

In animals inoculated with swIAV (both swIAV and swIAV/PRRSV groups), the viral genome was detected in nasal secretions at D4 and D7 post-inoculation, with significantly lower detection levels at D7 (Additional file 2A). At the time of PRRSV inoculation on D7, swIAV was detected in BALF from all the animals in the swIAV group, in lung tissue in two out of five pigs, and in the tracheobronchial lymph nodes in five out of five pigs. By D10 and D14, the swIAV genome was no longer detected in the nasal secretions of pigs in the swIAV/PRRSV group (data not shown). However, it was still detected in BALF in three out of five animals at D10 and in two out of five animals at D14 (Additional file 2B). In lung tissue, swIAV genome was detected in one out of five animals at D10 and D14. In TBLN, it was detected in three out of five animals at D10, but no more at D14 (Additional files 2C, D).

PRRSV genetic material was detected at D10 (3 days post-PRRSV infection) in all animals from the PRRSV and swIAV/PRRSV groups, in BALF, tracheobronchial lymph nodes and sera. In the lung tissue, PRRSV was detected in all the pigs from the swIAV/PRRSV group but in only two out of five animals in the PRRSV group. By D14 (7 days post-PRRSV infection), PRRSV was detected in all animals and all sample types (Figure 3A–D). However, no significant differences were observed between the PRRSV and swIAV/PRRSV groups at D10 or D14.

Thus, virological monitoring indicated that PRRSV loads in the lungs, lymph nodes and blood were not influenced by swIAV pre-infection.

Beyond the comparison between swIAV/PRRSV and PRRSV infections, we evaluated the correlation between PRRSV load and lung lesion scores. While PRRSV load in BALF, LN, and serum showed no significant correlation with lesion scores (data not shown), PRRSV lung load were positively correlated with both the Jung score (Figure 3E, r = 0.66, *p* = 0.015) and the Composite score (Figure 3F, r = 0.43, *p* = 0.056). Interestingly, the two lesion scores did not correlate with each other (Figure 3G) emphasizing their complementarity in assessing lung pathology.

### Interleukin-12 increased trend upon swIAV and swIAV/PRRSV infections in BALF

The cytokine profile (GM-CSF, IFN-γ, IL-10, TNF-α, IL-1α, IL-1β, IL-6, IL-8, IL-18, and IL-12) in BALF was assessed on D7 for the Mock and swIAV groups, and on D10 (3 days post-PRRSV inoculation) and D14 (7 days post-PRRSV inoculation) for the PRRSV and swIAV/PRRSV groups (Figure 4).

The levels of GM-CSF, IFN-γ, IL-10, and TNF-α were below detection thresholds. No significant differences were observed for the cytokines IL-1α, IL-1β, IL-8, and IL-18 (Figure 4A, B, D, E). In the swIAV group on D7, a significant increase in the pro-inflammatory cytokines IL-6 and IL-12 (a pro-Th1 cytokine) was observed compared to the Mock group (*p* < 0.01) (Figures 4C, F). On D10, a trend suggested higher IL-12 levels in the swIAV/PRRSV group compared to the PRRSV group, with four out of five animals of the PRRSV group showing lower concentrations than those in the swIAV/PRRSV group, and five out of five compared to the Mock group, although this difference was not statistically significant. This trend continued on D14, with all five animals in the swIAV/PRRSV group exhibiting higher IL-12 concentrations than those in the Mock group. The swIAV/PRRSV group presented a significant IL-12 overexpression when compared with the Mock group using Kruskal–Wallis unpaired comparisons test (Additional file 1). However, two out of five animals in the PRRSV group displayed IL-12 levels similar to those in the swIAV/PRRSV group.

### Pre-infection with swIAV increased number of conventional type 1 dendritic cells (cDC1) in BALC during PRRSV infection

To characterize the cellular innate immune response in more detail, mononuclear phagocyte cell populations were examined in BALC for all groups.

Conventional DC1 were defined as MHCII^high^CD11c^+^CD163^−^CD172a^−/low^CD1^−^, while cDC2 were defined as MHCII^high^CD11c^+^CD163^−^CD172a^+^CD1^+^. Monocyte-derived DC (moDC) were identified as MHCII^high^CD11c^+^CD163^low^CD172a^+^CD1^−^, and macrophages from the BALC were characterized as MHCII^high^CD11c^+^CD163^high^CD172a^+^ (Additional file 3).

In the Mock and swIAV groups at D7, no differences in the number of identified cell populations were observed (Figures 5A–D). Similarly, no significant differences were observed between the PRRSV and swIAV/PRRSV groups on D10 and D14, except for a significant increase in cDC1 on D14 in the swIAV/PRRSV group compared to the PRRSV group (*p* < 0.01) (Figure 5C). This increase in cDC1 remains significant when analyzed using the Kruskal–Wallis unpaired comparisons test (Additional file 1).

### Pre-infection with swIAV increased activated CD4^+^ regulatory and conventional CD4 T cells in BALC

To further explore the immune response, lymphoid cell populations in the BALC were analyzed by flow cytometry for all groups and all necropsy time points.

One set of experiments focused on conventional CD4^+^Foxp3^−^ T cells (Tconv) and CD4^+^Foxp3^+^ Treg cells (Figure 6A–D, Additional file 4), investigating their distribution within total CD4 T cells. No significant differences were observed in the percentages of Tconv and Treg between the swIAV and Mock groups, or between the PRRSV and swIAV/PRRSV groups (Figures 6A, B). Within the two subsets of Tconv and Treg, we also analyzed the co-expression of Inducible T-cell Costimulator (ICOS), involved in anti-inflammatory signalling and Ki-67, a molecule expressed in active stages of the cell cycle. A significant increase in ICOS^+^Ki-67^+^ Tconv and Treg cells was observed in the swIAV group compared to the Mock group. For the ICOS^+^Ki-67^+^ Tconv, this increase remained on D10 in the swIAV/PRRSV compared to the PRRSV group (Figure 6C). Interestingly, swIAV/PRRSV group presented a significantly higher frequency of ICOS^+^Ki-67^+^ Tconv cells when compared to the Mock group using Kruskal–Wallis unpaired comparisons test (Additional file 1). Moreover, a significant difference in ICOS^+^Ki-67^+^ Tconv cells was observed at D10 when comparing swIAV/PRRSV to the PRRSV group alone. Overall, this suggested that the swIAV infection resulted in an increase in activated Tregs and Tconv in the BALC that was maintained even in the context of a subsequent PRRSV infection.

### Swine IAV infection resulted in sustained increases in effector lymphocytes in BALC

A third flow cytometry panel focused on potential changes in conventional T cells, unconventional T cells and NK cells. Given the high numbers of addressed markers (n = 12, CD2, CD3, CD4, CD8α, CD8β, CD16, CD161, NKp46, Perforin, PLZF, T-bet, TCR-γδ) we performed dimensionality reduction using t-SNE clustering on live lymphocytes (Figures 7A–E, Additional file 5). This analysis enabled the identification of natural killer (NK) cells, conventional CD4 and CD8 T cells, and γδ T cells. The t-SNE analysis was set to perform a clustering for 12 clusters (Additional file 6, Additional file 7).

A number of clusters showed significant changes related to treatment groups. In the swIAV group, a significant increase compared to the Mock group was detected for clusters 6, 8, 9 and 10. Cluster 6 contained cells reminiscent of conventional CD4 T cells due to a CD3^+^CD4^+^CD8α^±^ phenotype that was negative for all other markers (Figure 7A). Cluster 8 consisted of cells with a phenotype of cytotoxic CD8 T cells (CD2^+^CD3^+^CD8αβ^+^perforin^±^, Figure 7C). Interestingly, cluster 9 contained cells that were negative for all lineage markers (CD3, TCR-γδ, CD4, CD8β), including NK associated markers NKp46 and CD16 but contained high levels of perforin (Figure 7D). Additionally, swIAV infection also led to an increase of cells represented by cluster 10 which consisted of CD3^+^ T cells that were negative for CD4, CD8β and TCR-γδ, but CD2^+^CD8α^+^perforin^dim^ (Figure 7E). Clusters 6, 8, 9 and 10 also presented a significant increase in swIAV/PRRSV group on D10 compared with Mock D7 when using Kruskal–Wallis unpaired comparisons test (Additional file 1). Notably, these elevated populations remained consistently higher in the swIAV/PRRSV groups at D14, showing significant differences compared to the PRRSV group (*p* < 0.05). An increase in a subset of TCR-γδ cells (cluster 7, Figure 7B) with a CD8α^+^CD2^±^perforin^±^ phenotype was also observed in the swIAV/PRRSV group compared to PRRSV group on D14.

To explore the correlations between the different variables involved in responses to PRRSV in the context of swIAV pre-infection or not, we performed a PCA, which included PRRSV loads in the lung (Figure 3B) along with all the other variables depicted in Figures 4, 5, 6, 7, encompassing the data from PRRSV-infected and swIAV/PRRSV-infected animals (D10 PRRSV, D10 swIAV/PRRSV, D14 PRRSV, D14 swIAV/PRRSV groups). The first two PCA dimensions (Dim) accounted for 29% (Dim1) and 18% (Dim2) of the data complexity respectively. As opposed to Dim 1, Dim 2 distinctly segregated PRRSV-infected from swIAV/PRRSV-infected animals (Figure 8A). Notably, variables primarily represented by Dim1 (Cos2 > 0.3) included the Composite lung lesion scores associated with lung PRRSV load, moDC, cDC2, Treg ICOS^+^/Ki67^+^ and Tconv ICOS^+^/Ki67^+^ BALC as well as IL-12 and IL-1β serum concentrations (Figure 8B). Conversely, Dim2 was associated (Cos2 > 0.3) with Clusters 6 to 10, cDC1 and AM BALC variables.

### Pre-infection with swIAV increased IFN-γ-producing CD4 T cells after PRRSV re-stimulation

We investigated IFN-γ and IL-2 production in T cells within PBMCs following in vitro re-stimulation with the autologous PRRSV-1 strain by intracellular cytokine staining. Analyses focused on the PRRSV and swIAV/PRRSV groups, isolated on D14 (7 days post-PRRSV infection) and the Mock group. Cells cultured in medium alone served as negative controls. CD4 expressing lymphocytes were pre-gated and cytokine producing cells separated into CD8α^+^ and CD8α^−^ subsets (Additional file 7).

**Figure 9:**
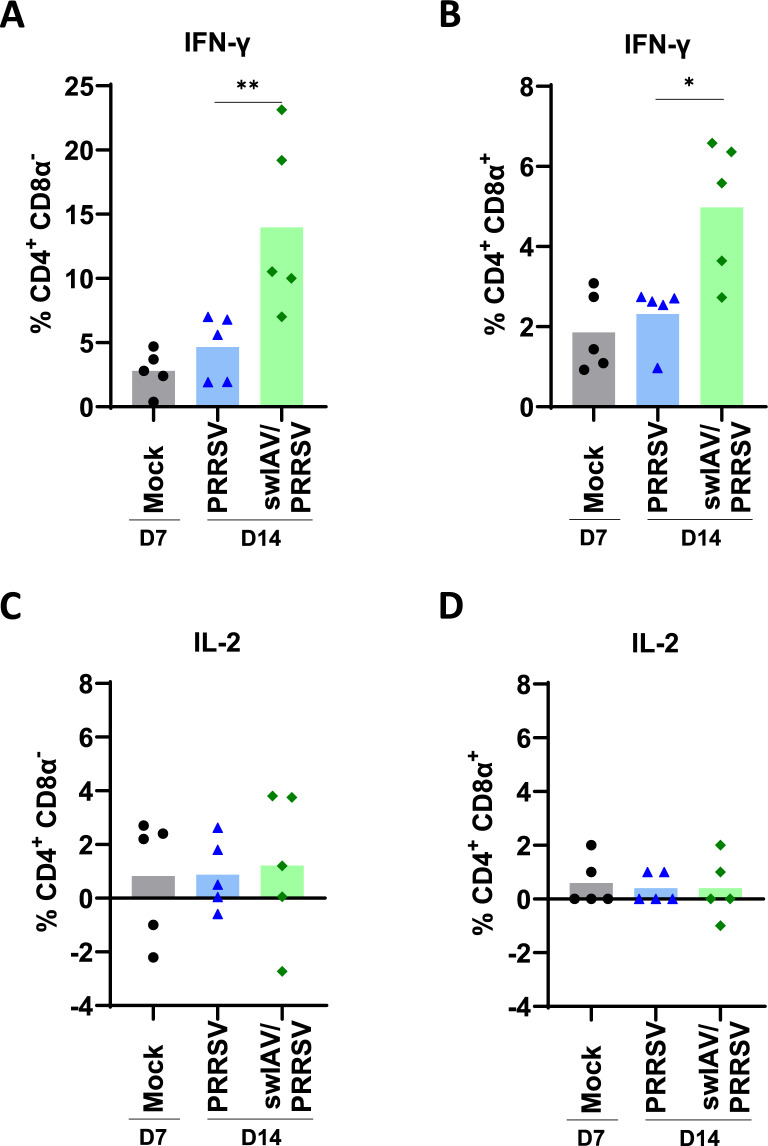
**PRRSV-specific CD4 T cell responses in PBMC.** **A**–**F** The percentages of IFN-γ and IL-2 producing cells within total CD4 T cells were analyzed in PBMC isolated at D14. Cells were restimulated in vitro for 18 h with PRRSV (MOI 1) or cultured with medium. **A**, **B** IFN-γ. **C**, **D** IL-2. The percentages were calculated by subtracting the percentage of cytokine-producing cells cultured with medium only). Values are obtained by subtracting the percentage obtained in the live PRRSV restimulation condition from the percentage obtained in the unstimulated condition. Statistical analysis was performed using the Mann–Whitney unpaired, non-parametric test, (*) *p* < 0.05 or (**) *p* < 0.01 (mean; *n* = 5).

Irrespective of CD8α expression, the percentage of IFN-γ-producing CD4 T cells was significantly higher in the swIAV/PRRSV group on D14 compared to PRRSV groups (*p* < 0.05) (Figure 8A, B) and the Mock group when using Kruskal–Wallis unpaired comparisons test (Additional file 1). Notably, the percentages in the PRRSV group remained at levels comparable to those observed in the Mock group (Figure 9A, B). No significant differences were observed in the production of IL-2 (Figure 9C, D). Of note, the IFN-γ response in PBMCs induced by this PRRSV strain typically begins around 2 weeks after inoculation.

### Local swIAV antibodies continued to rise after swIAV clearance

Humoral immune responses to swIAV and PRRSV infections were also assessed by detecting specific antibodies directed against each virus. For animals infected with swIAV, IgA in BALF and IgG in both BALF and serum were detected (Additional files 8A-C). S/P ratios continued to rise on D10 and D14, although most pigs had cleared swIAV by those time points, which is typical in single swIAV infections (Additional file 2).

No significant differences in anti-PRRSV IgA and IgG antibody levels were observed in the BALF of the PRRSV and swIAV/PRRSV groups throughout the study (Additional files 9A, B). At D14, 7 days after PRRSV inoculation, IgA and IgG antibodies began to be detectable in some animals. Similarly, no significant differences in anti-PRRSV IgG and IgM levels were observed in serum between the PRRSV and swIAV/PRRSV groups. The S/P ratios remained low but started to increase at D14 (Additional file 9C, D).

## Discussion

Co-infections with different pathogens are often the cause of respiratory diseases in pigs [1, 2]. It is established that infection with IAV increases susceptibility to secondary bacterial infections, such as *Streptococcus suis* in pigs [15, 29, 30] or *Streptococcus pneumoniae* in humans [16, 19, 31]. One of the proposed mechanisms for this influenza-related susceptibility is the depletion of AM, as suggested from studies in mice between 4 and 10 days post-IAV inoculation [17–19]. In our study, we did not observe differences in the number of AM in animals from the swIAV group compared to the Mock group at 7 days post-IAV inoculation. This suggests that, differently to IAV infection in mice, the swIAV strain used in this study did not lead to AM depletion in pigs. However, it is important to approach the analysis of resident AM during IAV infection with caution. During this infection various chemokines have increased expression in the lungs, leading to a heterogeneous influx of innate immune cells, such as monocytes-derived macrophages (moMΦ) and moDC [17–19, 32], whose surface markers might vary based on inflammatory conditions. It would therefore be interesting to consolidate these results in our study on pigs using multiparametric, unbiased approaches, such as single-cell RNA sequencing, to more precisely distinguish resident cells from recruited ones.

While various combinations of viral infections are possible, our study focused on a PRRSV-1 super-infection occurring 1 week after a swIAV H1N2 infection, both commonly observed on pig farms in France. The objective of our research was to evaluate the impact of a primary swIAV infection on the host’s immune responses during a secondary PRRSV infection. We analyzed the effects on clinical parameters, viral loads, and both innate and adaptive immune responses in pigs.

One week after the initial infection with swIAV, clinical signs following the secondary infection with PRRSV did not differ significantly from those observed during a single infection with PRRSV. This suggested that the initial swIAV infection did not exacerbate the animals' health status during the subsequent PRRSV infection. Intriguingly, PRRSV load was more variable in lung tissue compared to BALF, TBLN and serum. The PRRSV load in lung, but not in the other tissues, was directly correlated with lung lesion score. We may speculate that lung tissue viral load directly accounts for in situ viral replication and the resulting inflammation, leading to lesions. Moreover, the lung tissue is composed of multiple cell types; most of them are not susceptible to PRRSV while BALC is composed of 90% of AM, the main PRRSV target cell type. Thus, the higher proportion of target cells in the BALC may explain both the higher viral load and the higher (intra-group) homogeneity of the results in these samples compared to lung tissue. Conversely, viral load in TBLN and serum shall result from viral replication in lung and other tissues and their spill over in these compartments resulting in a more homogeneous viral load.

It is noteworthy that in a previous study using the same swIAV H1N2 and PRRSV-1 strains, but with a reversed order of infection (PRRSV inoculation followed by swIAV inoculation 8 days later), Bougon et al. [14] observed a reduction in clinical signs in the co-infected group, suggesting an attenuation of the impact of swIAV H1N2 infection in pigs previously infected with PRRSV-1. Correlation analyses revealed an association between IFN-α production and the onset of clinical signs. PRRSV infection led to a reduction in IFN-α production in PRRSV/swIAV super-infected pigs, consistent with previous observations [33], which likely contributed to the attenuation of clinical signs and the pro-inflammatory response induced by the influenza infection [14]. They also reported a transient yet significant decrease in PRRSV load in the lungs of super-infected pigs, which was correlated with the induction of IFN-α by swIAV, a cytokine to which PRRSV is particularly sensitive [34]. Another study by Renson et al. [35] examined the effects of H1N2 swIAV infection on the replication of a PRRSV-1 modified live vaccine (MLV1) in SPF piglets. SwIAV infection 6 h before MLV1 administration delayed MLV1 viremia and post-vaccination seroconversion. The early rise in IFN-α levels following H1N2 swIAV infection likely explained the inhibition of MLV1 replication. These results highlighted that the order of infections may play a key role in modulating clinical responses. By contrast, in this study, the animals were super-infected 7 days after the swIAV infection, when IFN-α was no longer detectable in BALF [14], to avoid any interference from this cytokine.

The main objective of the present study was to evaluate the effect of a primary infection with swIAV on the early progression of a subsequent PRRSV infection. No significant differences were observed in the PRRSV load in the lungs and blood of pigs super-infected with swIAV and PRRSV compared to those infected only with PRRSV.

We also aimed to examine whether swIAV could induce an early immune imprint, similar to what has been observed in the murine IAV [22] and adenovirus [21] models. Aegerter et al. [22] showed that primary infection with IAV conferred protection against *Streptococcus pneumoniae* by recruiting AM derived from monocytes, which persisted in the lungs for an extended period following IAV infection. Similarly, adenovirus promoted a lasting innate immune memory of AM, facilitated by T lymphocytes through the production of IFN-γ, enhancing immunity against *S. pneumoniae* and *E. coli* [21]. It is important to note that in these studies, the protective imprint was observed several weeks after IAV inoculation (at least 4 weeks), while in this experiment, PRRSV infection was performed only 1 week after swIAV inoculation. However, the present results did not show that primary infection with swIAV offered protection against PRRSV or on contrary enhanced its replication in the early stages following swIAV infection.

Interestingly, the PCA revealed two distinct sets of variables. The first set, associated with lung lesions and PRRSV lung load, included serum IL-12 and IL-1β, as well as BALC moDC, cDC2, and conventional and regulatory ICOS⁺Ki67⁺ CD4 T cells. In a previous study, we linked moDC (also referred to as inflammatory DCs) to IL-1β production in pigs [32], while moDCs have also been implicated in pathogenic anti-influenza immune responses [30]. Conversely, porcine and murine cDC2 [32, 36] have been associated with Th2-biased responses. This may have contributed to the increase in activated CD4 Treg cells. Porcine ICOS^+^Ki-67^+^Foxp3^−^ CD4 T cells (Tconv) likely comprise multiple functional subsets, making their role more difficult to interpret. Nevertheless, our data suggest an association between PRRSV load, lung lesions, local moDC and a potential Th2 microenvironment.

The second set of variables strongly linked the swIAV pre-infection group to a higher BALC content of AM, cDC1, and lymphocyte clusters 6 to 10. Given the complexity of sampling and the challenge of implementing interventional studies in swine, establishing a direct cause-and-effect relationship among these cell types remains difficult. However, it is conceivable that XCR1-positive cDC1 cells were recruited to the lung by CD8⁺ T and NK-related XCL1-producing lymphocytes [37–39]. Alternatively, cDC1 may activate and drive the expansion of T perforin-expressing T and NK-related subsets (clusters 7–10) [32, 40]. This coincided with a PRRSV-specific Th1 response in the blood of pigs that were initially infected with swIAV. These results suggest that a primary swIAV infection could promote the rapid induction of an anti-PRRSV-1 immunity via the recruitment of cDC1.

It is well established that PRRSV infection leads to a delayed activation of PRRSV-specific CD4 and CD8 T cells, typically appearing 2 to 3 weeks post-inoculation [14, 41]. However, the present study indicated that prior infection with swIAV increased the presence of CD4 and perforin expressing lymphocytes in the lungs during PRRSV infection, potentially contributing to a more pronounced immune response against PRRSV. It remains to be determined whether this increase in effector cells was due to active recruitment of new cells to the lungs or the persistence of cells initially activated by the swIAV infection, but their increase in swIAV-only infected pigs suggests the latter. Indeed, CD4, CD8, and γδ T lymphocytes were present in the BALC as early as 6 days post-infection with swIAV [42], and swIAV-specific cells could be detected between D4 and D7 in PBMCs [43, 44]. However, this proliferation of lymphocytes did not prevent PRRSV replication at the early stages of infection, and their role in controlling PRRSV at later stages remains to be clarified.

A longer-term follow-up of PRRSV infection would be valuable to determine if it is better controlled over time through these cellular responses. Additionally, it would be relevant to explore whether enhanced induction of type-1 responses could contribute to protection against a subsequent homologous or heterologous PRRSV challenge.

In summary, this study demonstrated that the primary infection with swIAV, occurring 1 week prior, did not interfere with the early infection of AM by PRRSV. However, several parameters of the cellular antiviral response were significantly elevated, indicating that an enhanced immune response could influence the progression of PRRSV infection at later stages. These results raise questions about the potential impact of this response on the progression of PRRS disease. Therefore, further research is needed to better understand the underlying mechanisms of these immune responses.

## Supplementary Information

**Additional file 1.** **Statistical results of the Kruskal-Wallis test significant for the different figures.**

**Additional file 2.** **swIAV genomic load in nasal swab, BALF, lung tissue, and tracheobronchial lymph nodes.** (A-D) Quantification of swIAV genomic loads by RT-qPCR in (A) nasal swab, (B) BALF, (C) lung tissue, and (D) lymph nodes (mean ± SD; *n* = 5-10). For nasal swab statistical analysis was performed using the Mann-Whitney unpaired non-parametric test. (**) *p* < 0.01, (***) *p* < 0.001, (****) *p* < 0.0001 (mean ± SD; *n* = 5-10).

**Additional file 3.** **Gating strategy for mononuclear phagocyte staining**. Alveolar macrophages, type 1 and type 2 conventional dendritic cells (cDC1 and cDC2, respectively), and monocyte-derived DCs (moDCs) were identified in BAL. Mononuclear phagocyte populations were defined using MHC II/CD11c/CD163/CD172a/CD1 markers.

**Additional file 4.** **Gating strategy for regulatory and CD4 conventional T lymphocyte staining. **Regulatory T cells (Treg) and CD4 conventional T cells (Tconv) were identified in BAL. T CD4 cells were defined using CD3/CD4/CD8α markers, with Treg cells further distinguished by FoxP3/CD25 expression. Subsequently, Treg and Tconv subpopulations were identified based on ICOS/Ki-67 expression.

**Additional file 5.** **Proportions of lymphoid cell populations in BAL.** (D-1) Relative expression levels of CD2, CD3, CD4, CD8α, CD8β, and CD16 for each animal group within clusters are represented by color, ranging from high (red) to low (blue). (D-2) Relative expression levels of CD161, NKp46, perforin, T-bet, and TCR-γδ for each animal group within clusters are represented by color, ranging from high (red) to low (blue).

**Additional file 6.** **Proportions of lymphoid cell populations in BAL.** (A-H) Live lymphocytes from BAL were clustered using the t-SNE algorithm, with clusters 1-5 and 11-12 presented. Statistical analysis was performed using the Mann-Whitney unpaired non-parametric test. (*) *p* < 0.05, (**) *p* < 0.01 (mean; *n* = 5).

**Additional file 7.** **Gating strategy for intracellular cytokine staining.** Conventional CD4 T cells were identified in PBMC. CD4 T cells were defined using CD4/CD8α markers. IFN-γ, TNF-α, and IL-2 producing cells within CD4^+^ and CD8α^+/-^ T cells were identified.

**Additional file 8.** **Anti-swIAV antibodies in BALF and serum.** (A) Levels of IgA and (B) IgG (NP protein) in BALF. (C) Anti-IgG levels in serum (mean ± SD; *n* = 5).

**Additional file 9.** **Anti-PRRSV antibodies in BALF and serum.** (A) Levels of IgA and (B) IgG (N protein) in BALF. (C) Anti-IgG and IgM levels in serum (mean ± SD; *n* = 5).

## Data Availability

The datasets used and/or analysed during the current study are available from the corresponding author on reasonable request.
