# Supplementary material for: Swine influenza A virus infection sets the local immunological landscape in subsequent infection with porcine reproductive and respiratory syndrome virus
Source: Vet Res. 2025 Jun 8;56:114. doi: 10.1186/s13567-025-01536-6 (PMC12147356; doi:10.1186/s13567-025-01536-6)
Supplement: Supplementary file 1 — Additional file 1. Statistical results of the Kruskal-Wallis test significant for the different figures. [file 13567_2025_1536_MOESM1_ESM.pptx]

## Slide 1
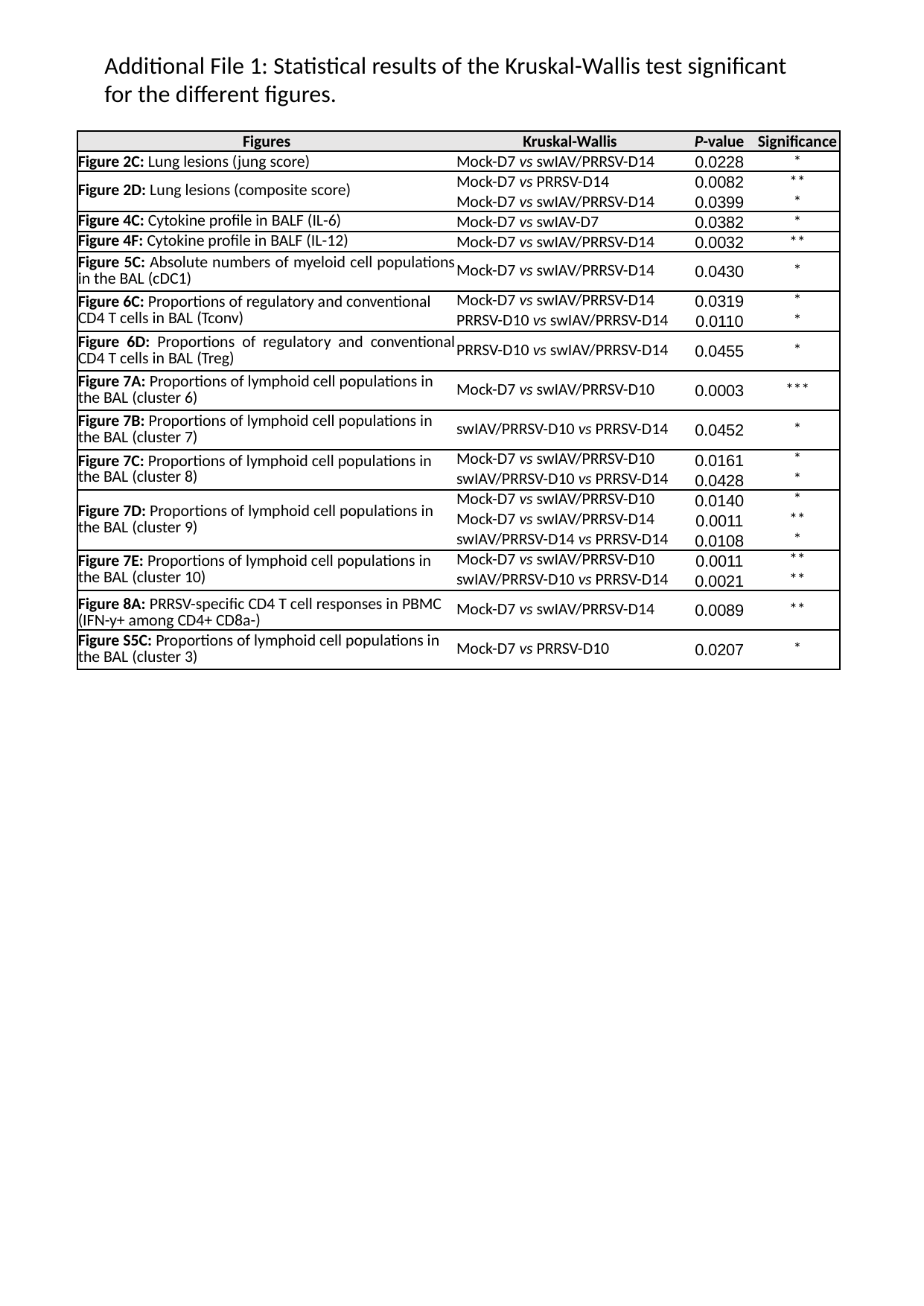

Additional File 1: Statistical results of the Kruskal-Wallis test significant for the different figures.
| Figures | Kruskal-Wallis | P-value | Significance |
| --- | --- | --- | --- |
| Figure 2C: Lung lesions (jung score) | Mock-D7 vs swIAV/PRRSV-D14 | 0.0228 | \* |
| Figure 2D: Lung lesions (composite score) | Mock-D7 vs PRRSV-D14 | 0.0082 | \*\* |
| | Mock-D7 vs swIAV/PRRSV-D14 | 0.0399 | \* |
| Figure 4C: Cytokine profile in BALF (IL-6) | Mock-D7 vs swIAV-D7 | 0.0382 | \* |
| Figure 4F: Cytokine profile in BALF (IL-12) | Mock-D7 vs swIAV/PRRSV-D14 | 0.0032 | \*\* |
| Figure 5C: Absolute numbers of myeloid cell populations in the BAL (cDC1) | Mock-D7 vs swIAV/PRRSV-D14 | 0.0430 | \* |
| Figure 6C: Proportions of regulatory and conventional CD4 T cells in BAL (Tconv) | Mock-D7 vs swIAV/PRRSV-D14 | 0.0319 | \* |
| | PRRSV-D10 vs swIAV/PRRSV-D14 | 0.0110 | \* |
| Figure 6D: Proportions of regulatory and conventional CD4 T cells in BAL (Treg) | PRRSV-D10 vs swIAV/PRRSV-D14 | 0.0455 | \* |
| Figure 7A: Proportions of lymphoid cell populations in the BAL (cluster 6) | Mock-D7 vs swIAV/PRRSV-D10 | 0.0003 | \*\*\* |
| Figure 7B: Proportions of lymphoid cell populations in the BAL (cluster 7) | swIAV/PRRSV-D10 vs PRRSV-D14 | 0.0452 | \* |
| Figure 7C: Proportions of lymphoid cell populations in the BAL (cluster 8) | Mock-D7 vs swIAV/PRRSV-D10 | 0.0161 | \* |
| | swIAV/PRRSV-D10 vs PRRSV-D14 | 0.0428 | \* |
| Figure 7D: Proportions of lymphoid cell populations in the BAL (cluster 9) | Mock-D7 vs swIAV/PRRSV-D10 | 0.0140 | \* |
| | Mock-D7 vs swIAV/PRRSV-D14 | 0.0011 | \*\* |
| | swIAV/PRRSV-D14 vs PRRSV-D14 | 0.0108 | \* |
| Figure 7E: Proportions of lymphoid cell populations in the BAL (cluster 10) | Mock-D7 vs swIAV/PRRSV-D10 | 0.0011 | \*\* |
| | swIAV/PRRSV-D10 vs PRRSV-D14 | 0.0021 | \*\* |
| Figure 8A: PRRSV-specific CD4 T cell responses in PBMC (IFN-y+ among CD4+ CD8a-) | Mock-D7 vs swIAV/PRRSV-D14 | 0.0089 | \*\* |
| Figure S5C: Proportions of lymphoid cell populations in the BAL (cluster 3) | Mock-D7 vs PRRSV-D10 | 0.0207 | \* |
