# Supplementary figures and images for: Swine influenza A virus infection sets the local immunological landscape in subsequent infection with porcine reproductive and respiratory syndrome virus
Source: Vet Res. 2025 Jun 8;56:114. doi: 10.1186/s13567-025-01536-6 (PMC12147356; doi:10.1186/s13567-025-01536-6)

## Slide 1
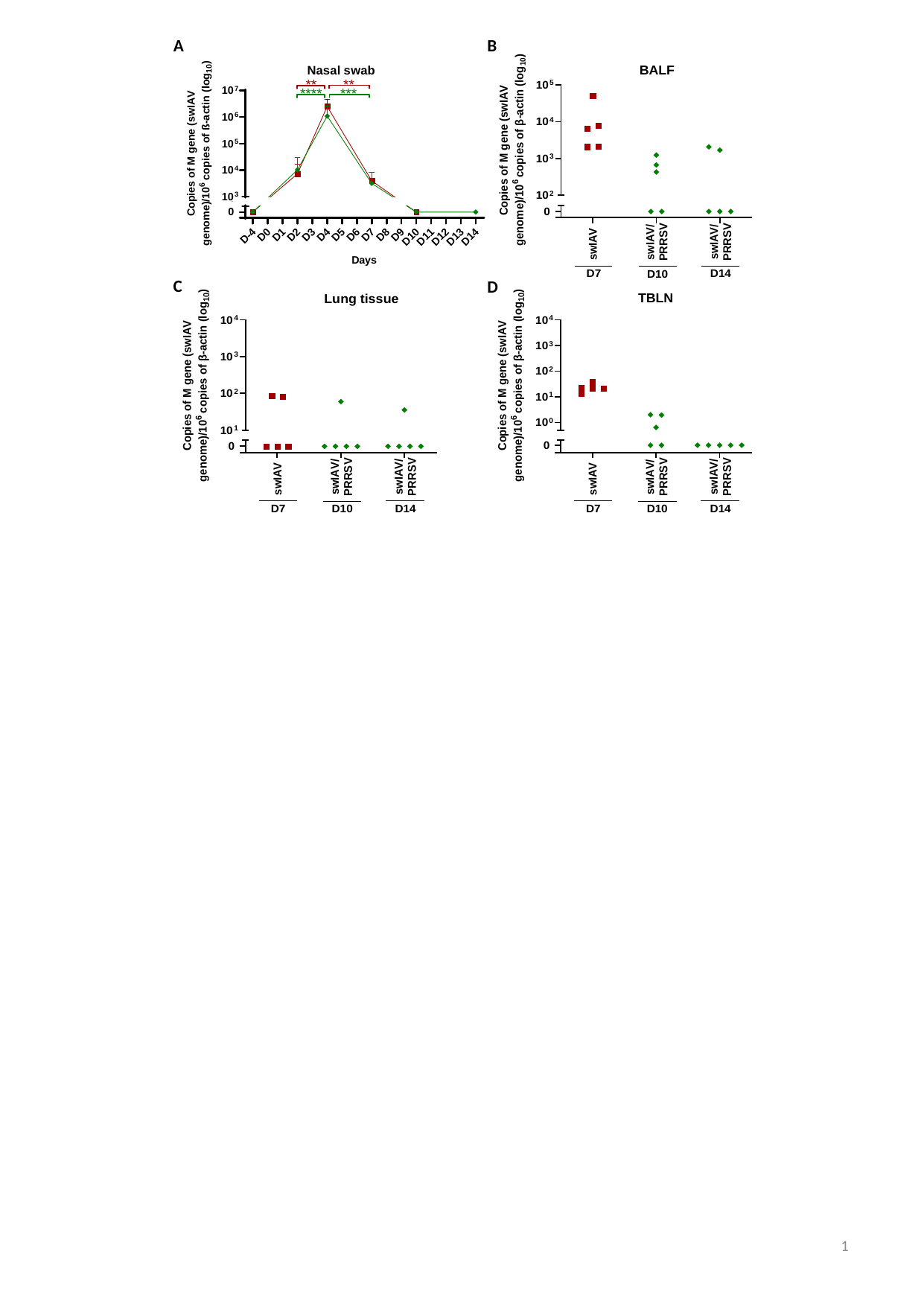

A
B
C
D
1

Supplement: Supplementary file 2 — Additional file 2. swIAV genomic load in nasal swab, BALF, lung tissue, and tracheobronchial lymph nodes. (A-D) Quantification of swIAV genomic loads by RT-qPCR in (A) nasal swab, (B) BALF, (C) lung tissue, and (D) lymph nodes (mean ± SD; n = 5-10). For nasal swab statistical analysis was performed using the Mann-Whitney unpaired non-parametric test. (**) p < 0.01, (***) p < 0.001, (****) p < 0.0001 (mean ± SD; n = 5-10). [file 13567_2025_1536_MOESM2_ESM.pptx]

## Slide 1
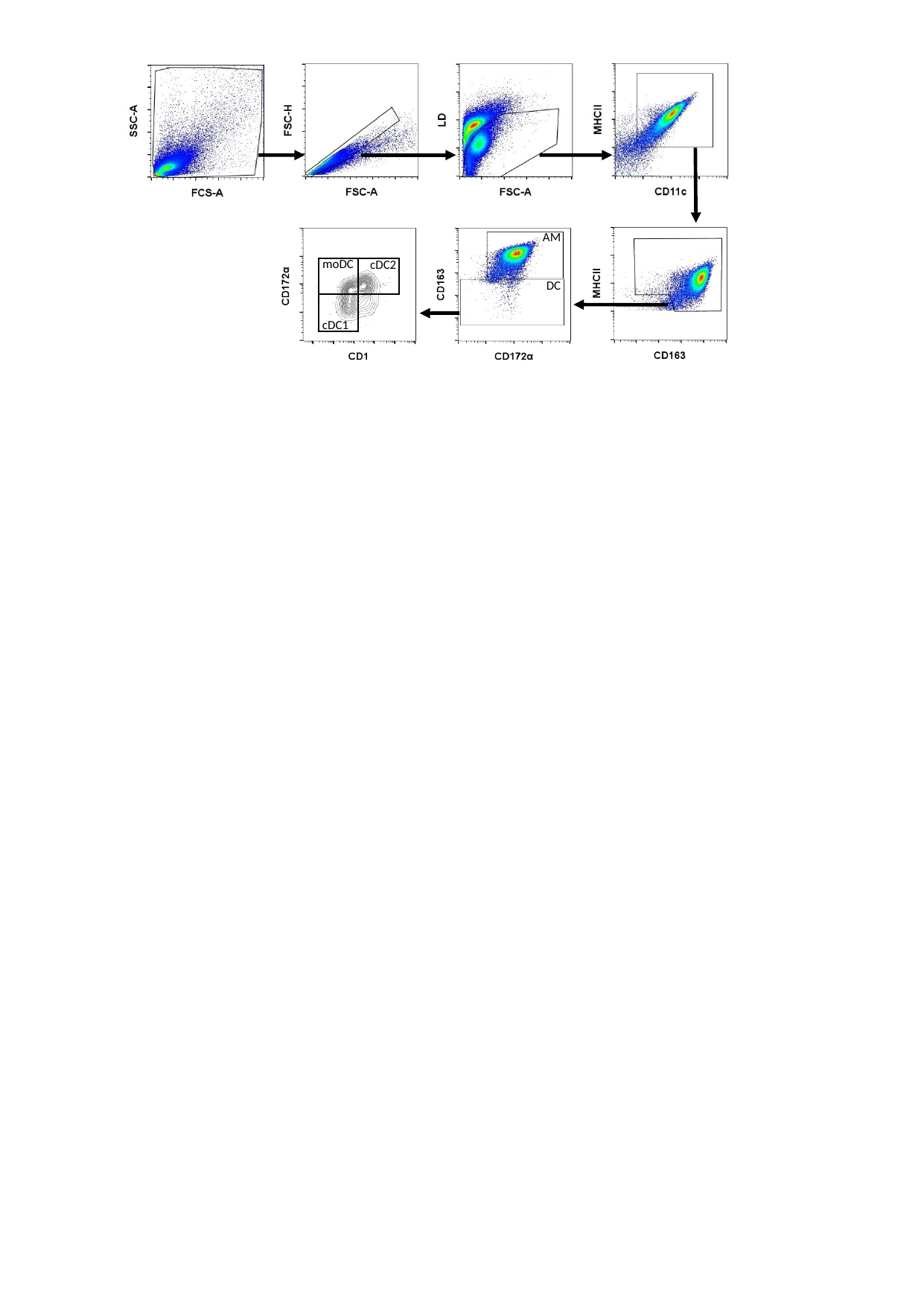

moDC
cDC2
cDC1
AM
DC

Supplement: Supplementary file 3 — Additional file 3. Gating strategy for mononuclear phagocyte staining. Alveolar macrophages, type 1 and type 2 conventional dendritic cells (cDC1 and cDC2, respectively), and monocyte-derived DCs (moDCs) were identified in BAL. Mononuclear phagocyte populations were defined using MHC II/CD11c/CD163/CD172a/CD1 markers. [file 13567_2025_1536_MOESM3_ESM.pptx]

## Slide 1
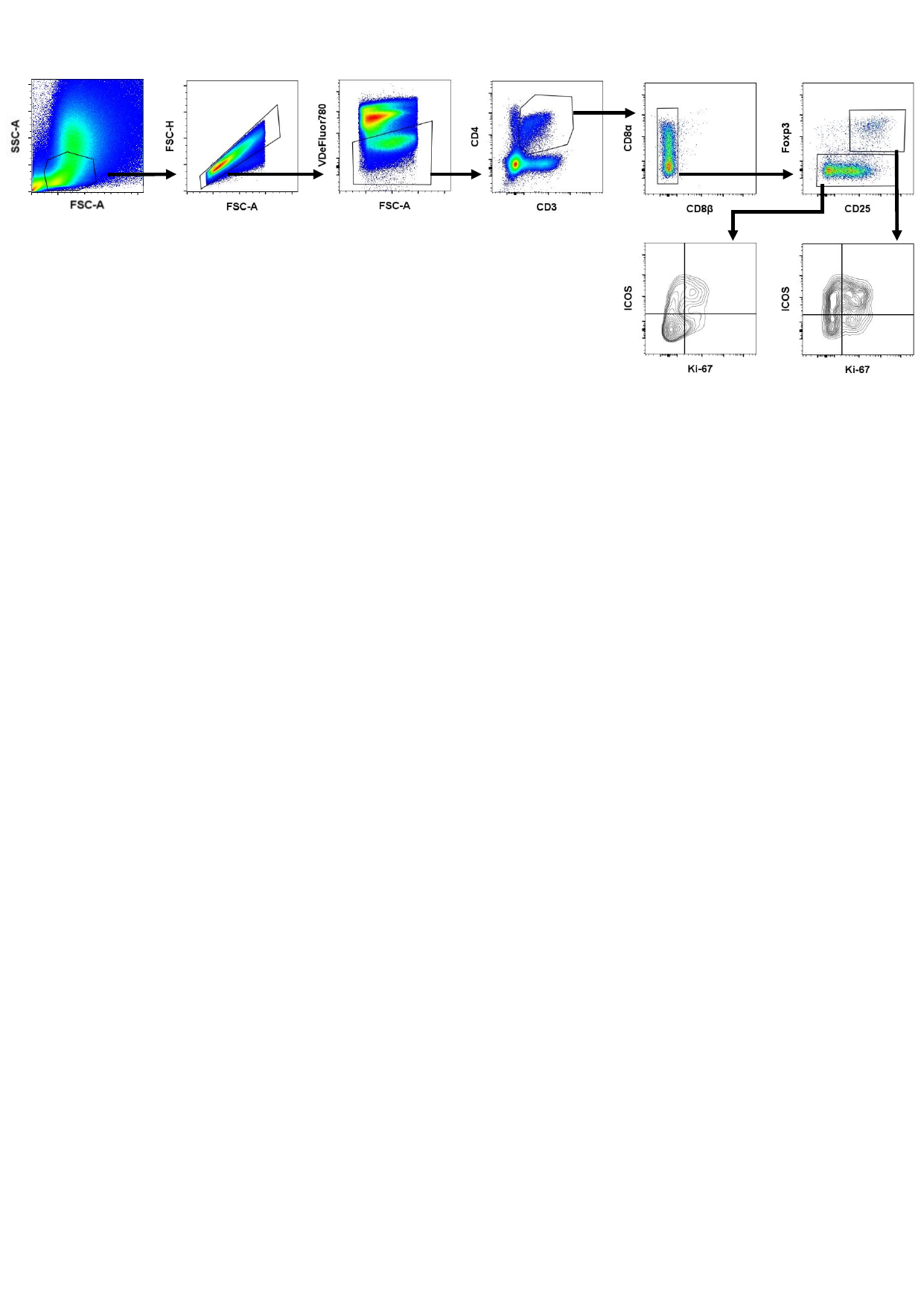

Supplement: Supplementary file 4 — Additional file 4. Gating strategy for regulatory and CD4 conventional T lymphocyte staining. Regulatory T cells (Treg) and CD4 conventional T cells (Tconv) were identified in BAL. T CD4 cells were defined using CD3/CD4/CD8α markers, with Treg cells further distinguished by FoxP3/CD25 expression. Subsequently, Treg and Tconv subpopulations were identified based on ICOS/Ki-67 expression. [file 13567_2025_1536_MOESM4_ESM.pptx]

## Slide 1
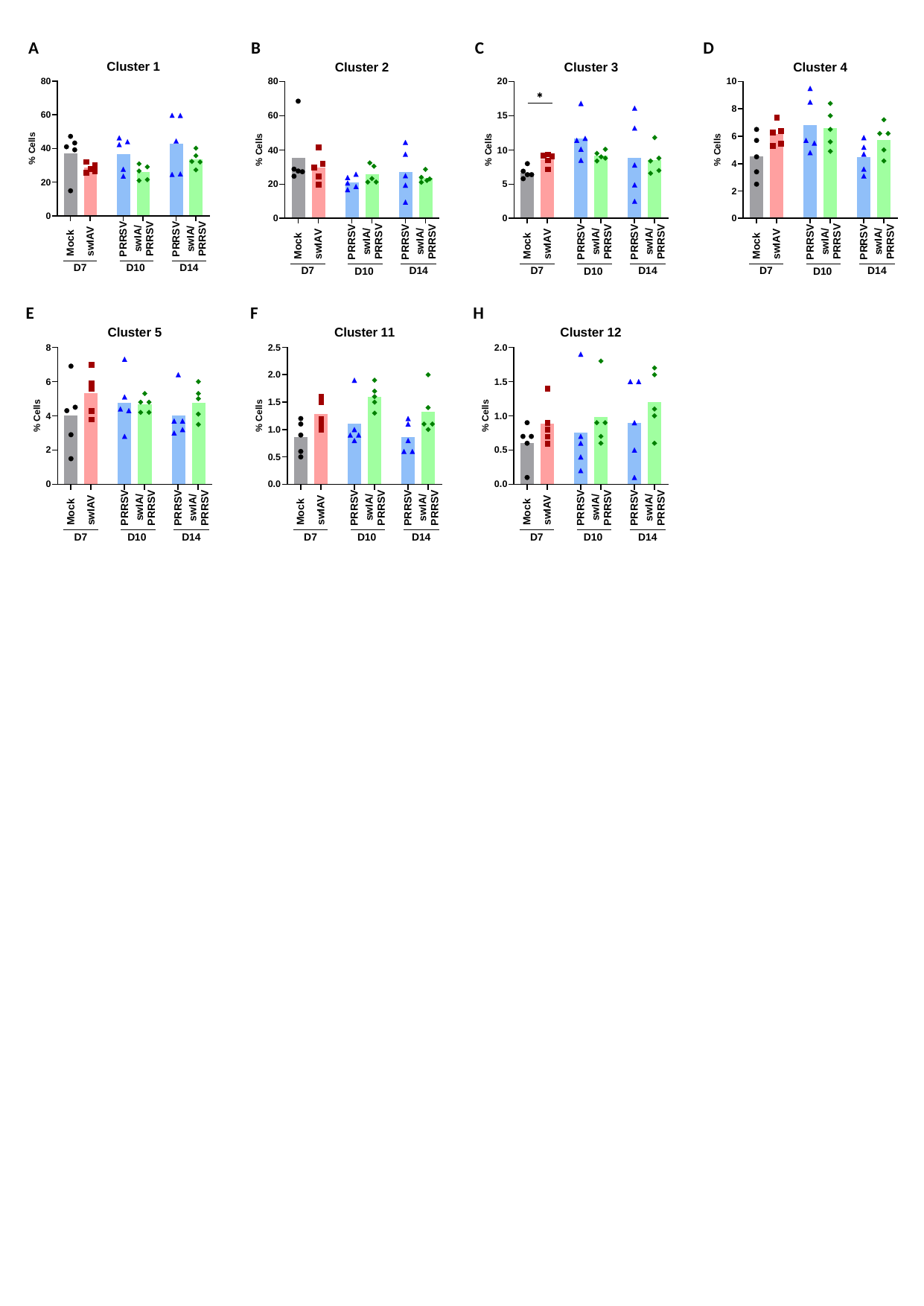

B
A
C
E
F
D
H

Supplement: Supplementary file 6 — Additional file 6. Proportions of lymphoid cell populations in BAL. (A-H) Live lymphocytes from BAL were clustered using the t-SNE algorithm, with clusters 1-5 and 11-12 presented. Statistical analysis was performed using the Mann-Whitney unpaired non-parametric test. (*) p < 0.05, (**) p < 0.01 (mean; n = 5). [file 13567_2025_1536_MOESM6_ESM.pptx]

## Slide 1
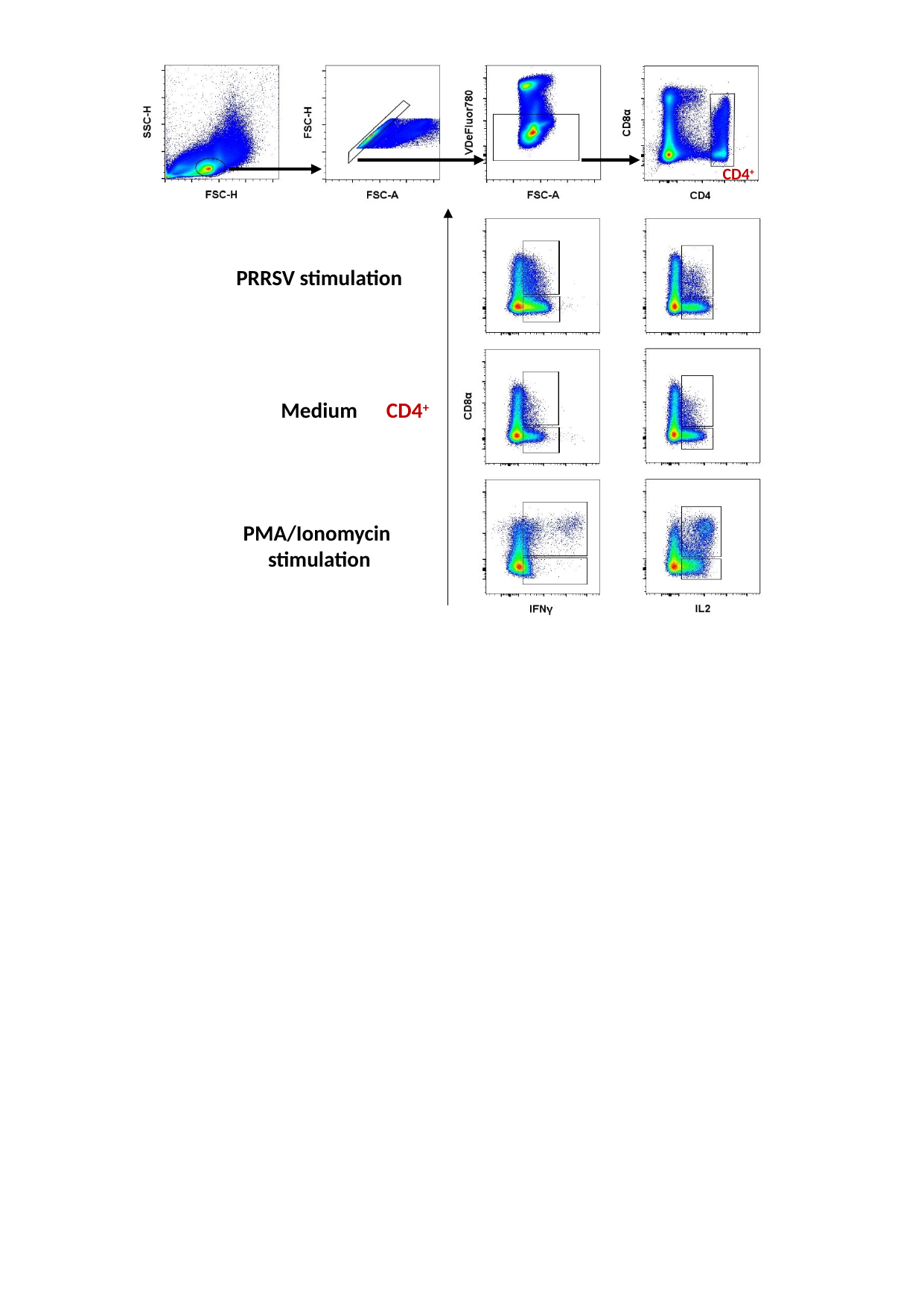

CD4+
PRRSV stimulation
Medium
CD4+
PMA/Ionomycin
stimulation

Supplement: Supplementary file 7 — Additional file 7. Gating strategy for intracellular cytokine staining. Conventional CD4 T cells were identified in PBMC. CD4 T cells were defined using CD4/CD8α markers. IFN-γ, TNF-α, and IL-2 producing cells within CD4+ and CD8α+/- T cells were identified. [file 13567_2025_1536_MOESM7_ESM.pptx]

## Slide 1
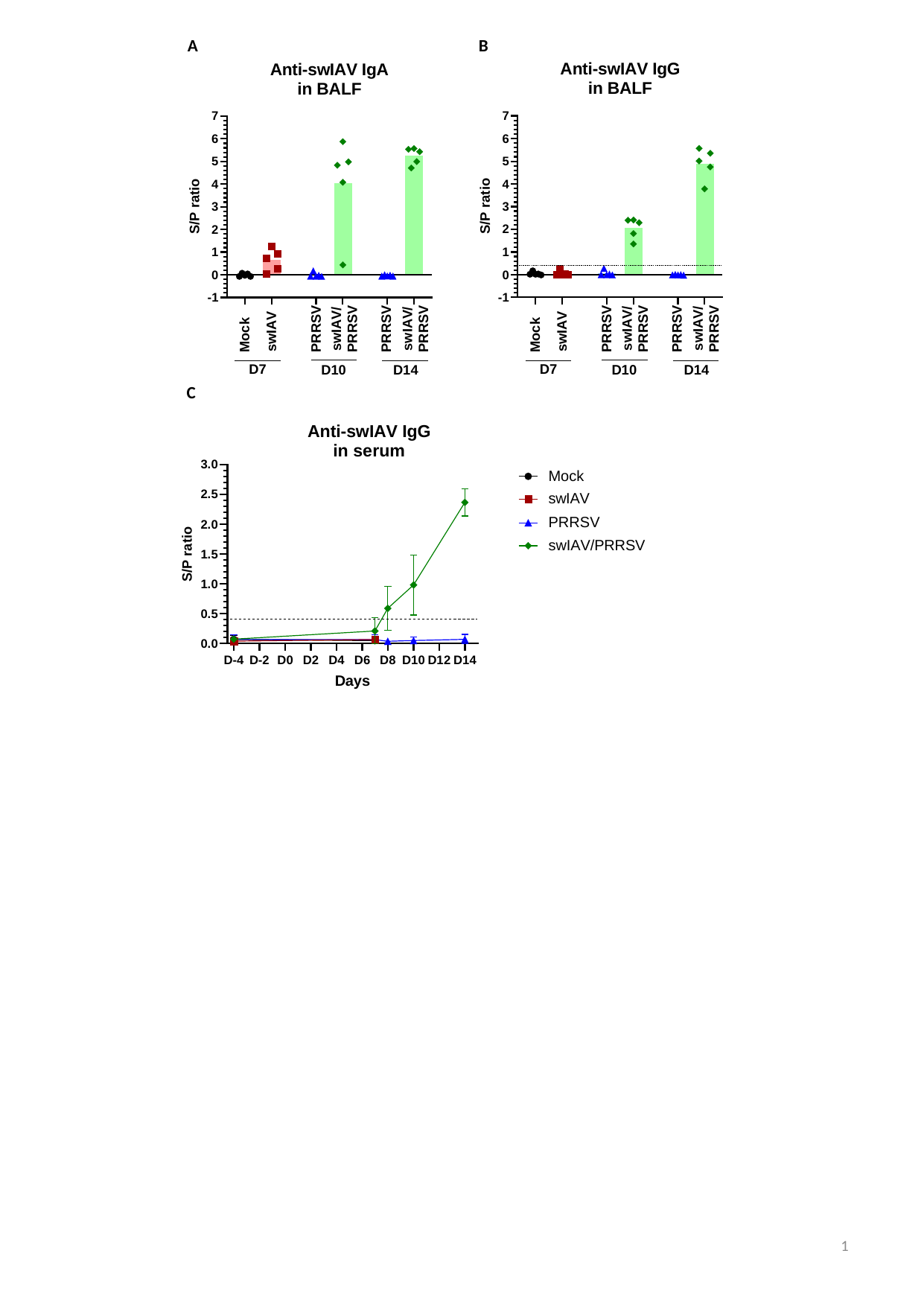

A
B
C
1

Supplement: Supplementary file 8 — Additional file 8. Anti-swIAV antibodies in BALF and serum. (A) Levels of IgA and (B) IgG (NP protein) in BALF. (C) Anti-IgG levels in serum (mean ± SD; n = 5). [file 13567_2025_1536_MOESM8_ESM.pptx]

## Slide 1
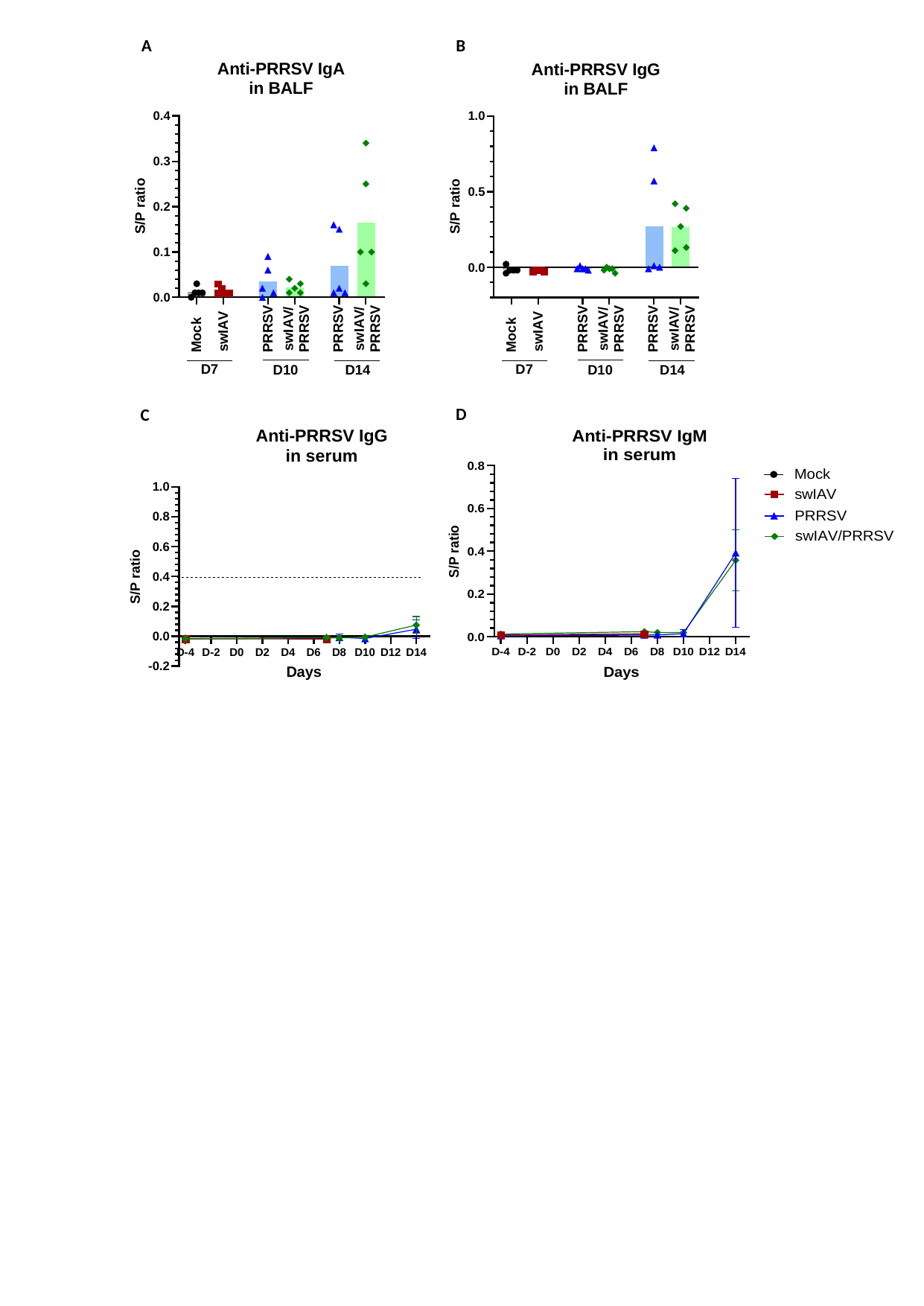

A
B
D
C

Supplement: Supplementary file 9 — Additional file 9. Anti-PRRSV antibodies in BALF and serum. (A) Levels of IgA and (B) IgG (N protein) in BALF. (C) Anti-IgG and IgM levels in serum (mean ± SD; n = 5). [file 13567_2025_1536_MOESM9_ESM.pptx]
