# Supplementary material for: Swine influenza A virus infection sets the local immunological landscape in subsequent infection with porcine reproductive and respiratory syndrome virus
Source: Vet Res. 2025 Jun 8;56:114. doi: 10.1186/s13567-025-01536-6 (PMC12147356; doi:10.1186/s13567-025-01536-6)
Supplement: Supplementary file 5 — Additional file 5. Proportions of lymphoid cell populations in BAL. (D-1) Relative expression levels of CD2, CD3, CD4, CD8α, CD8β, and CD16 for each animal group within clusters are represented by color, ranging from high (red) to low (blue). (D-2) Relative expression levels of CD161, NKp46, perforin, T-bet, and TCR-γδ for each animal group within clusters are represented by color, ranging from high (red) to low (blue). [file 13567_2025_1536_MOESM5_ESM.pptx]

## Slide 1
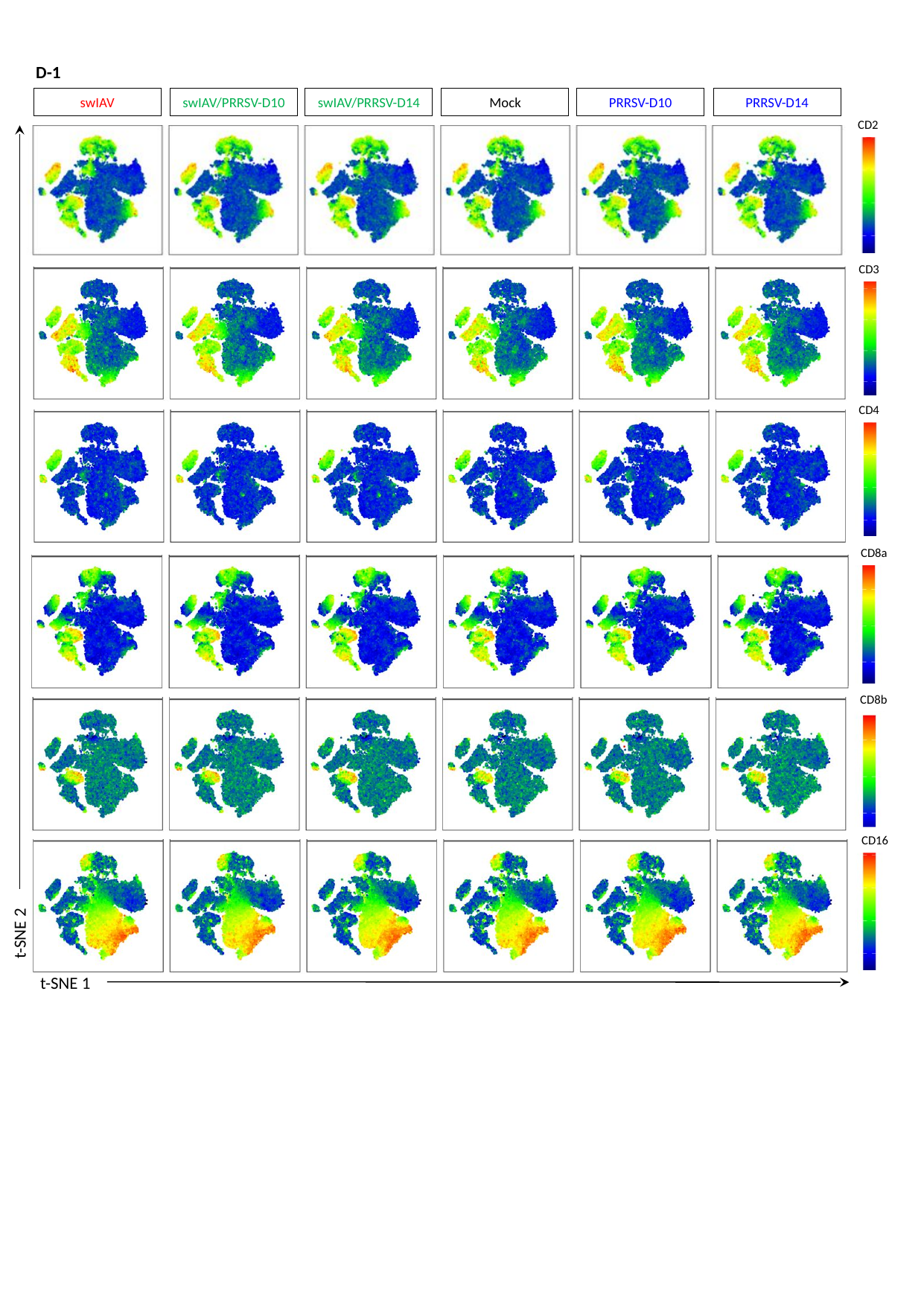

D-1
swIAV
swIAV/PRRSV-D10
swIAV/PRRSV-D14
Mock
PRRSV-D10
PRRSV-D14
CD2
CD3
CD4
CD8a
CD8b
CD16
t-SNE 2
t-SNE 1

## Slide 2
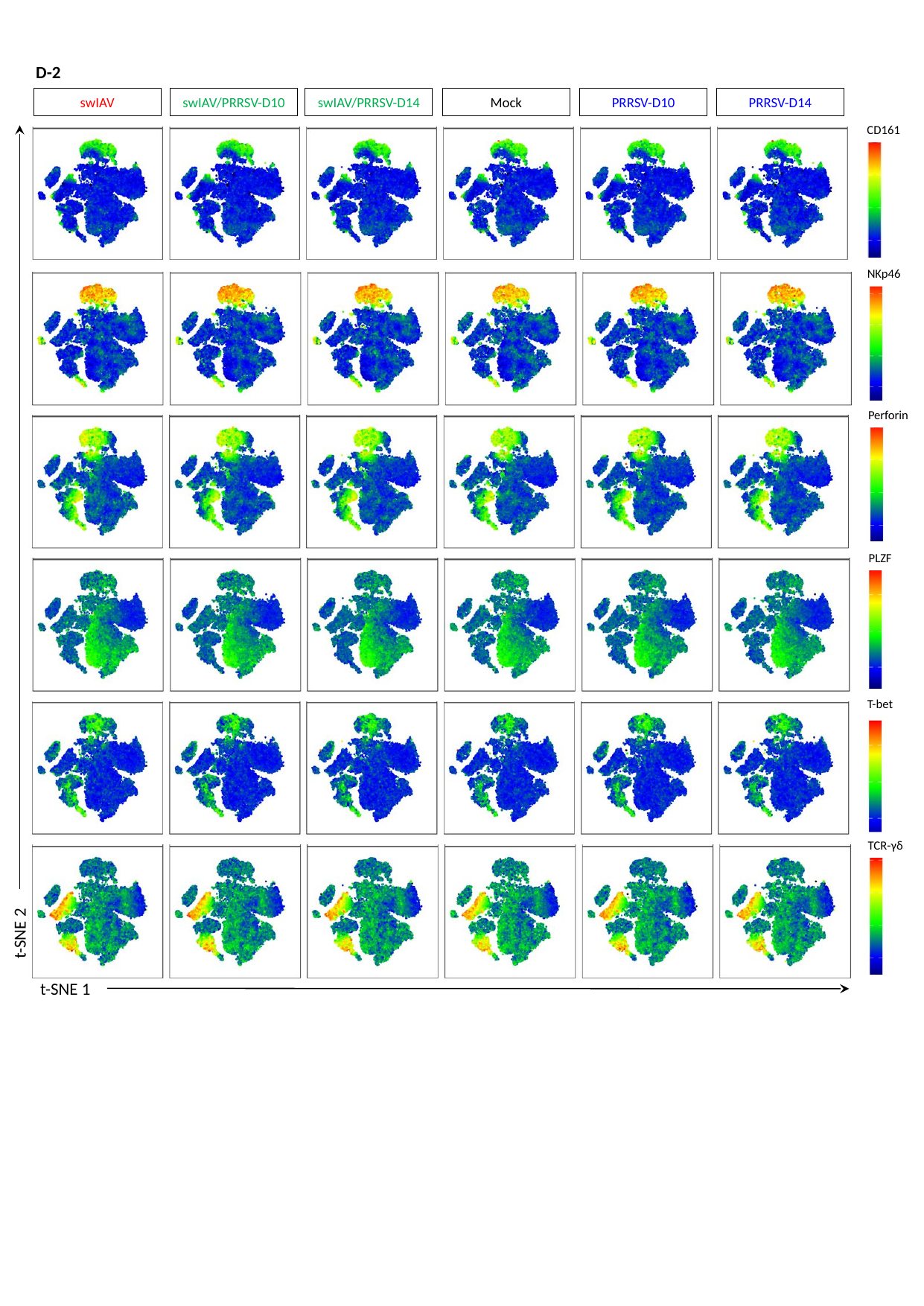

D-2
swIAV
swIAV/PRRSV-D10
swIAV/PRRSV-D14
Mock
PRRSV-D10
PRRSV-D14
CD161
NKp46
Perforin
PLZF
T-bet
TCR-γδ
t-SNE 2
t-SNE 1
